# Assessing the efficiency of multiple sequence alignment programs

**DOI:** 10.1186/1748-7188-9-4

**Published:** 2014-03-06

**Authors:** Fabiano Sviatopolk-Mirsky Pais, Patrícia de Cássia Ruy, Guilherme Oliveira, Roney Santos Coimbra

**Affiliations:** 1 Center for Excellence in Bioinformatics, Centro de Pesquisas René Rachou (CPqRR), Fundação Oswaldo Cruz (FIOCRUZ/Minas), Belo Horizonte, MG Brazil; 2 Genomics and Computational Biology Group, CPqRR, Instituto Nacional de Ciência e Tecnologia, FIOCRUZ/Minas, Belo Horizonte, MG Brazil; 3 Biosystems Informatics, CPqRR, FIOCRUZ/Minas, Avenida Augusto de Lima 1715. Barro Preto, Belo Horizonte, MG Brazil

**Keywords:** Multiple sequence alignment, Computer programs, Accuracy, Performance

## Abstract

**Background:**

Multiple sequence alignment (MSA) is an extremely useful tool for molecular and evolutionary biology and there are several programs and algorithms available for this purpose. Although previous studies have compared the alignment accuracy of different MSA programs, their computational time and memory usage have not been systematically evaluated. Given the unprecedented amount of data produced by next generation deep sequencing platforms, and increasing demand for large-scale data analysis, it is imperative to optimize the application of software. Therefore, a balance between alignment accuracy and computational cost has become a critical indicator of the most suitable MSA program. We compared both accuracy and cost of nine popular MSA programs, namely CLUSTALW, CLUSTAL OMEGA, DIALIGN-TX, MAFFT, MUSCLE, POA, Probalign, Probcons and T-Coffee, against the benchmark alignment dataset BAliBASE and discuss the relevance of some implementations embedded in each program’s algorithm. Accuracy of alignment was calculated with the two standard scoring functions provided by BAliBASE, the sum-of-pairs and total-column scores, and computational costs were determined by collecting peak memory usage and time of execution.

**Results:**

Our results indicate that mostly the consistency-based programs Probcons, T-Coffee, Probalign and MAFFT outperformed the other programs in accuracy. Whenever sequences with large N/C terminal extensions were present in the BAliBASE suite, Probalign, MAFFT and also CLUSTAL OMEGA outperformed Probcons and T-Coffee. The drawback of these programs is that they are more memory-greedy and slower than POA, CLUSTALW, DIALIGN-TX, and MUSCLE. CLUSTALW and MUSCLE were the fastest programs, being CLUSTALW the least RAM memory demanding program.

**Conclusions:**

Based on the results presented herein, all four programs Probcons, T-Coffee, Probalign and MAFFT are well recommended for better accuracy of multiple sequence alignments. T-Coffee and recent versions of MAFFT can deliver faster and reliable alignments, which are specially suited for larger datasets than those encountered in the BAliBASE suite, if multi-core computers are available. In fact, parallelization of alignments for multi-core computers should probably be addressed by more programs in a near future, which will certainly improve performance significantly.

## Background

The objectives of Multiple Sequence Alignment (MSA) are manifold, the most important being: detecting key functional residues, predicting secondary or tertiary structures and inferring the evolutionary history of a protein family. Several programs and algorithms have been developed over time for sequence alignment. Given the unprecedented amount of data produced by next generation deep sequencing platforms, large-scale data resources are emerging. Therefore, the alignment accuracy and computational costs of MSA programs are critical indicators of the most suitable program for each particular dataset. Finding the correct balance between speed and accuracy can be tricky, especially without objective parameters to enable direct comparison.

The pairwise alignment of two sequences can be performed with two different approaches: global or local. Global alignments attempt to align entire sequences, up to both ends of each sequence [1]. Local alignments attempt to identify subsequences sharing high similarity [2]. Although dynamic programming guarantees a mathematically optimal alignment of sequences, heuristics-based algorithms are preferred as they require less computational capacity, suitable in studies involving multiple sequences. The vast majority of heuristics-based MSA programs align sequences using the progressive approach, combining global and/or local methods [3]. This type of algorithm builds a MSA through a series of consecutive pairwise alignments, following the branching order of a guide tree. The progressive method has the drawback that once errors are introduced at an early step, they cannot be removed later. One of the first MSA programs combining progressive and global pairwise alignment is CLUSTALW [4].

A successful improvement of the progressive alignment is the adoption of a consistency approach. Consistency maximizes the agreement of pairwise alignments and, therefore, tries to avoid mistakes in the progressive MSA. This issue is illustrated as follows: for any three sequences 1, 2 and 3, the pairwise alignment of 1/2 and 2/3 implies an alignment of 1/3 that may be different from the direct alignment of 1/3. This motivates the search for an agreement with the set of pairwise alignments in order to obtain higher accuracy of alignment in MSA. The progressive MSA programs DIALIGN-TX [5] and T-Coffee [6] are consistency-based, however the former uses local pairwise alignments, whereas the later uses both global and local pairwise alignments. Probcons [7] and Probalign [8] use a *probabilistic consistency transformation* step to incorporate multiple sequence conservation information during pairwise alignment, thus providing information that can be used to guide the progressive alignment. Probcons and CLUSTAL OMEGA [9] incorporates a pair-Hidden Markov Model-based algorithm (HMM), and Probalign computes a partition function as a substitution to the HMM implementation. Alternatively, Lee and co-workers [10] developed the Partial Order Alignment algorithm (POA), in which nucleotides or amino acids are represented as a linear series of nodes, each node connected by a single incoming and a single outgoing edge. The graph representation of an MSA, that can itself be aligned directly by pairwise dynamic programming, guarantees that the optimal alignment between each pair of sequences will be considered.

As alignment errors may occur in any progressive MSA, post-processing steps such as iterative refinement [11] may correct some miss-alignments. That is the case of the progressive programs MUSCLE [12] and MAFFT [13]. The iterative refinement steps implemented in these programs are based on a technique called tree-dependent restricted partitioning [14]. In this strategy, a MSA is partitioned into two groups, which are later re-aligned. The process is repeated until no true quality improvements are made. Probcons and Probalign also adopt an iterative refinement step. MAFFT, which can incorporate a consistency step during the iterative refining method [15], also introduces Fast Fourier Transform (FFT) in sequence alignment. In FFT, an amino acid sequence is transformed into a sequence composed of volume and polarity values for each residue, allowing for the rapid detection of homologous segments. Since substitutions between physically and chemically similar amino acids tend to preserve the protein structure, a neutral substitution of similar amino acids would keep key residues correctly aligned.

The efficiency of MSA programs can benchmarked, resulting in useful guidelines. In order to assess and compare the efficiency of the nine programs listed above, the BAliBASE benchmark dataset was selected [16]. BAliBASE was the first large scale benchmark specifically designed for MSA, providing high quality manually refined reference alignments based on 3D structural superpositions. The benchmark presents challenging test cases simulating real problems faced when aligning multiple sequences, which are divided into different datasets. The current version of the BAliBASE is divided into several reference datasets. The first five contain: Reference 1- cases with small numbers of equidistant sequences, and was further subdivided by percent identity; Reference 2- families with one or more “orphan” sequences; Reference 3- a pair of divergent subfamilies, with less than 25% identity between the two groups; Reference 4- sequences with large terminal extensions (N/C-terminal); and Reference 5- sequences with large internal insertions and deletions. For References 1 to 3 and 5, full-length sequences were provided in addition to the sequences with homologous regions only, to test the performance of MSA methods in the presence of “noise” in the form of non-conserved extensions. The next three reference alignments, 6, 7 and 8, contain sequences with repeats, transmembrane regions, and inverted domains, respectively. Reference 6 contains subsets with repeats having different residue similarity and input order and with the presence of additional domains. Reference 7 includes sequences with predicted transmembrane regions divided into subgroups with highly conserved core blocks. Reference 8 have sequences with two different domains in which their order is not preserved [17]. The most recent dataset added to BAliBASE is Reference 9 [18], which includes protein families with linear motifs often found in disordered regions that are difficult to align.

Although studies comparing the efficiency of some MSA programs through References 1 to 5 of the BAliBASE dataset are available [7,8,19-22], the remaining references have not been included in those benchmarks. In addition, the computational time required to complete the alignments and memory usage have not been systematically evaluated. We evaluated the alignment accuracy and computational cost of nine different multiple sequence alignment programs with the BAliBASE dataset and discuss the relevance of some implementations embedded in the programs algorithms. Reference 8 was not considered for this benchmark since comprises protein sequences that contain two different domains not in the same order in all homologues. For that, two independent alignments are provided by BAliBASE, one for each permuted domain. Once the selected MSA programs output one single alignment, where residues are kept in their input order, it is not possible to benchmark their performance.

## Methods

### Multiple sequence alignment programs

MSA programs were chosen based on different algorithmic approaches beyond download availability and popularity. All programs with their versions, URL for download and main algorithms are presented in Table 1. The programs were run using their default parameters for protein alignment with three exceptions: 1) MAFFT run in “auto” mode where, given the size of the dataset analyzed, the L-INS-i (iterative refinement with consistency from local pairwise alignment) method was mostly selected among the others; 2) POA run with the BLOSUM62 substitution matrix since there is no default matrix for this program and the input sequence order was preserved; 3) T-Coffee initially run in single-core mode and, when necessary, the multi-core parameter was adjusted to use all sixteen hardware processors as specified in “Computational cost assessment” section.

**Table 1:** Multiple sequence alignment programs used in this study

**Software**	**Download link**	**Main algorithm**
CLUSTALW v2.0.10	http://www.clustal.org/download/current/	Progressive
CLUSTAL O. v1.2.0	http://www.clustal.org/omega	Hidden Markov Model
DIALIGN-TX v1.0.2	http://dialign-tx.gobics.de/download	Consistency
MAFFT v6.714b	http://mafft.cbrc.jp/alignment/software/	Fast Fourier T./Iterative/Consistency
MUSCLE v3.8.31	http://www.drive5.com/muscle/downloads.htm	Iterative
POA v2	http://sourceforge.net/projects/poamsa	Graphs
Probalign v1.4	http://cs.njit.edu/usman/probalign/	Partition Function/Consistency
Probcons v1.12	http://probcons.stanford.edu/download.html	Hidden Markov Model/Consistency
T-Coffee v8.99	http://www.tcoffee.org/Projects_home_page/t_coffee_home_page.html	Consistency (multi-core usage capable)

### Hardware specifications

All programs were run on a DELL R900 Server with 4 Quad-Core E7430 @ 2.13 GHz, 8 MB Cache Memory, 8 × 8 GB of RAM and 2 TB HD.

### Benchmark dataset

Version 3.0 of the BAliBASE benchmark dataset is available at: ftp://ftp-igbmc.u-strasbg.fr/pub/BAliBASE3.

### Accuracy assessment

The scoring program Bali-score provided by BAliBASE assessed accuracy of each program. Two different scores estimate the accuracy of an alignment comparing to the BAliBASE reference alignment: the Sum-of-Pairs score (SP), and the Total-Column score (TC). All SP and TC scores were obtained by aligning full-length sequences and short truncated sequences, when available, with the nine programs.

The SP score determines the extent to which the programs succeed in aligning input sequences in an MSA. It is calculated as the ratio of the sum of scores *p* for all pairs of residues in every column of the alignment by the sum of scores in the reference alignment; *p*?=?1 if the pair of compared residues is aligned identically in the reference alignment, otherwise *p*?=?0. Thus, the SP score increases with the number of sequences aligned correctly.

The TC score is a binary score function which tests the ability of the programs to correctly align all sequences. The TC score is calculated considering the ratio of the sum of scores *c* by the number of columns in the alignment, being *c*?=?1 if all residues in the column are aligned identically in the reference alignment, otherwise *c*?=?0 [20].

In Reference set 9, a scoring program named Bali-score-elm was introduced. This program is a substitution to the previous one, estimating the accuracy of the alignment of the motif regions only. The Bali-score-elm program evaluates both SP and TC scores in true positive or false negative motifs regions, which can be aligned unambiguously in the region of the reference motif.

### Computational cost assessment: time of execution and peak memory usage

Perl and bash scripts (available upon request) were written in order to capture peak memory usage and execution time of alignments. Time of execution was collected after the complete alignment of each reference set. Peak memory usage was collected also for each aligned reference set.

**Figure 1:**
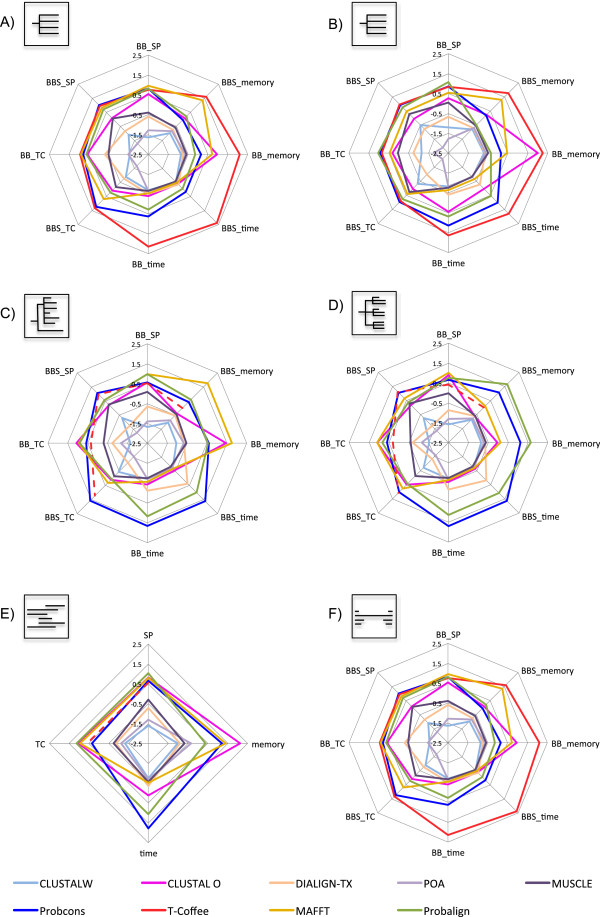
**Z-scores of SP, TC, memory and execution time measures for MSA programs in References 1–5.** Each of the six radar charts **(“A” to “F”)** represent one of the BAliBASE reference datasets (RV11, RV12, RV20, RV30, RV40 and RV50) respectively. The color lines represent the used MSA programs and dashed lines represent programs that exceed 2.5 hours of execution. The numbers represent the deviation pattern either positive, above the average, or negative, bellow the average of the programs.

T-Coffee run in single-core mode and, whenever the execution time exceeded two and a half hours in any of the eight datasets, the process was killed. Only then, parallelized T-Coffee run with sixteen cores and the peak memory and execution time were not compared with those of other programs (dashed lines in Figure 1 – C, D, E and Additional file 1 – C, F, H, M, N, O, P and Q). Probalign and Probcons also exceeded two and a half hours in the last three subsets of Reference set 9 (dashed lines in Additional file 1 – O, P and Q) and all six processes were killed.

### Statistical analysis

For each reference dataset, the average SP score, TC score and computational costs were obtained from the results produced by the nine MSA selected programs. Results are presented as Z-scores, meaning that for each accuracy scores and computational costs, the efficiency of the programs are expressed as the number of standard deviations, either positive or negative, from the average value. Then, the software GraphPad Prism version 5.02 (GraphPad Software, Inc., CA, USA) was used to test if the differences encountered in each measure by the programs were statistically significant. The one-way analysis of variance by ranks was done with the Friedman test, and pairs of groups were compared using the Dunn’s post-test. Differences were considered significant when *p* < 0.05.

## Results

### Alignment accuracy: sum-of-pairs and total-column scores

For Reference datasets 1 to 5, the accuracy of the alignments produced by Probcons, T-Coffee, Probalign, and MAFFT were consistently higher than that of the other programs (Figure 1). In fact, in these five reference test cases, all four programs had Z-scores above the average, being in some cases also statistically superior when compared to MUSCLE, CLUSTALW, CLUSTAL OMEGA, DIALIGN-TX and POA (See Additional file 1). When aligning available short versions of the sequences (BBS), Probcons and T-Coffee outperformed Probalign and MAFFT. Moreover, there was a statistically significant superiority of Probcons and T-Coffee in comparison to Probalign and MAFFT in References 1 and 2 (Additional file 1). When aligning full-length proteins (BB) of References 1 to 3 and 5, which represent more difficult test cases, and also Reference 4, where large terminal extensions are present, Probalign, MAFFT and, surprisingly, CLUSTAL OMEGA, generally outperformed both Probcons and T-Coffee. This switch in the top four scoring programs, regarding alignment of short truncated and full-length sequences, brought to our attention an interesting aspect of the performance of these programs. As seen in Additional file 2, for both SP and TC score values, the difference between aligning short sequences and full-length sequences was higher for Probcons and T-Coffee when compared to Probalign and MAFFT. The performance of CLUSTAL OMEGA was a bit contradicting. The program performed very well in three reference (Figure 1 – C, D and E) sets with full-length sequences but not with the short versions.

The remaining programs CLUSTALW, DIALIGN-TX and POA had Z-scores below the average in almost all test cases from the first five reference sets (Figure 1). MUSCLE was the only program presenting alignment accuracy values sometimes above the average, sometimes bellow. POA was the least accurate program when aligning truncated versions of the sequences, while CLUSTALW yielded the lowest accuracy for full-length sequences in almost all test cases.

For the remaining datasets of BAliBASE, all consistency-based programs, namely Probcons, T-Coffee, Probalign and MAFFT still produced alignments with higher accuracy when compared to the other five programs. CLUSTAL OMEGA presented excellent TC scores in some subsets of Reference 6. Still in Reference 6, Probalign SP and TC scores were superior, when compared to other programs, in several subsets (See Additional file 1 – A to G). MUSCLE presented improved results in the alignments produced at Reference 9 (See Additional file 1 – I to Q). In fact, MUSCLE generated alignments with higher SP and TC scores than MAFFT in some subsets (See Additional file 2 for more detailed scoring values).

### Computational cost: time of execution and peak memory usage

There is a general trade-off between computational cost and alignment accuracy in the entire BAliBASE dataset, as proposed before [20]. Our results indicated that CLUSTALW and MUSCLE were the fastest of the evaluated programs. CLUSTALW was also the program that consumed the least amount of memory, given the use of the efficient dynamic programming algorithm of Myers and Miller [23]. Among the programs that produced better SP and TC accuracy scores, as presented in the first section of the results, MAFFT was the fastest followed by CLUSTAL OMEGA. In fact, MAFFT was even faster than T-Coffee running in multi-core mode with just one exception at Reference 4. The drawback of MAFFT is that it requires more memory to run. The same happens for CLUSTAL OMEGA, at least when aligning full length sequences (BB) in the first five reference sets. As for T-Coffee running in single-core mode, results indicated that the program consumed generally more RAM than the others and was also the slowest in almost the entire reference sets. In several occasions, T-Coffee exceeded the threshold of 2.5 hours of execution time. Then, when running in multi-core mode, a significant gain in speed was observed, although memory usage was very high compared to other programs (more than 6 Gb of RAM consumed for subset RV931 from Reference 9). Probcons and Probalign also exceeded the 2.5 hours cutoff in the last three subsets from Reference 9 and, since no multi-core option is available, no alignments were provided for these subsets. For more details on execution time and memory usage see Additional file 3 and Additional file 4.

As for a direct correspondence of time of execution and memory usage, two major correlations were found. First, for all eight reference sets minus a few subsets of Reference 6, T-Coffee (single-core mode) had the highest values of memory usage and execution time to complete the alignments. Second, in the last subset of Reference 9, all programs which completed the alignments took more time and more memory usage to finish when compared to all their previous alignments. We excluded from this analysis: MAFFT, due to the “auto” mode selected parameter, and also T-Coffee running in multi-core mode. See Additional file 3 and Additional file 4 for more detailed comparisons.

## Discussion

Many MSA programs are freely available. However, choosing the most suitable program to each dataset is not trivial. The characteristics of the sequences to be aligned, such as the shared identity, as well as their number and length, are aspects that must be assessed in every MSA dependent project. Each MSA program parameterization, such as the choice of substitution matrices and gap opening/extending penalties for example, when available, also strongly affect the final alignment [24]. Running MSA programs with default parameters are usually preferred when no information regarding the sequences to be aligned are available and/or for users without previous knowledge in this particular field of sequence analysis. With that in mind, we chose to benchmark a selection of programs mostly with their default options. Although results presented herein are compatible with current low-cost hardware and timelines of most research projects, they must be used only as guidelines, and we encourage users to carefully study each program’s parameters in order to obtain the best possible output. The BAliBASE suite is a reliable benchmarking dataset, but still might be considered small to meet certain MSA projects [21]. Thus, understanding each programs own limitations are imperative in order to generate reliable results.

As stated in related papers [21,22], no available MSA program outperformed all others in all test cases. For the first five reference sets, our results indicated that T-Coffee, Probcons, MAFFT and Probalign were definitely superior with regard to alignment accuracy in all BAliBASE datasets, consistent with similar publications [7,8,21,22]. All four programs have a consistency-based approach in their algorithms, thus being a successful improvement in sequence alignment. Despite meeting certain consistency criteria, DIALIGN-TX is based on local pairwise alignments and is known to be outperformed by global aligners [5]. Nevertheless, we observed that the consistency-based approach may not offer alone the highest quality of alignment. CLUSTAL OMEGA did well when aligning some datasets with long N/C terminal ends from full-length sequences (BB) and has no consistency. The presence of these non-conserved residues at terminal ends, on the other hand, contributed to reduce the scores in the alignments generated by T-Coffee and Probcons, which produced the highest SP/TC scores when aligning the truncated sequences (BBS). Despite having an iterative refinement step, which could improve results, Probcons is still a global alignment program, thus being more prone to alignment errors induced by the presence of non-conserved residues at terminal ends [20]. Certainly MAFFT, Probalign and even CLUSTAL OMEGA may be preferred over T-Coffee and Probcons when aligning sequences with these long terminal extensions. The combination of iterative refinement strategy with consistency from local alignments in MAFFT (L-INS-i method) might have contributed to prevent and correct the alignment of the full-length sequences [22]. Similarly, the suboptimal alignments (determined by variations of the *Temperature* parameter) generated by the partition function of Probalign, might as well improved the ability of this program to deal with sequences with non-conserved terminal extensions [8]. Apparently, the profile HMM of long sequences also improved the alignments produced by CLUSTAL OMEGA.

As for the remaining reference sets of BAliBASE (6, 7 and 9), we observed that the four consistency-based programs mentioned above still generated better alignments, although MUSCLE presented improved results. In some subsets of Reference 9, MUSCLE was either close or better than some of the top four SP/TC scoring programs. At this reference set, the alignment of sequences with linear motifs generated by MUSCLE might be facilitated by *Kimura’s distance*, the second stage in the progressive alignment of this program. The *Kimura distance* states that only exact matches contribute to the match score. Although fast, the method has limitations since it does not consider which changes of amino acids are occurring between sequences. This limitation may be reverted in benefit since the program, assuming the same penalty for any amino acid substitution in early steps of progressive alignment, would avoid a distance increase between pairs of close sequences with errors or wildcard residues (any amino acid) at the linear motifs.

In the largest BAliBASE datasets, the use of the multi-core capability of T-Coffee was indispensable in order to evaluate alignment accuracy because, when running in single-core mode, its computational time exceeded by far the pre-established threshold of 2.5 hours. In the biggest dataset (the last subset of Reference 9), T-Coffee took more than nine days to complete the alignment. The parallelization of T-Coffee should certainly be seen as a major improvement to an MSA program, as processing cores are growing in number even in home desktop computers, not to mention more and faster RAM modules. Interestingly, MAFFT was the only program, among the top four SP/TC scoring programs, able to align all reference sets in less than 2.5 hours with the pre-established settings described in the Methodology section. This is most likely due to the flexibility of the “auto” mode of MAFFT to choose the most appropriate method of alignment according to dataset size, changing from high accuracy mode (L-INS-i) to high speed and less accuracy mode (FFT-NS-2) [25]. Although not being the version used in this work, recent improvements in parallelization were also achieved for MAFFT [26], indicating a tendency to make full use of available hardware and reduce time of execution of MSA programs. Besides parallelization, there is still much space for improvement in the field of multiple sequence alignment in performance. E.g., CLUSTAL OMEGA implemented a modified version of mBed [27], which produced fast and accurate guide trees, and managed to reduce computational time and memory requirements to finish the alignment of large datasets. A part from performance, there also much room for accuracy improvements, as some results presented in this study were still far from the BAliBASE reference alignments.

## Conclusions

Based on the results presented herein, all four consistency-based programs Probcons, T-Coffee, Probalign and MAFFT are well recommended for achieving better accuracy at multiple sequence alignments. Generally, the alignments of Probcons and T-Coffee were better than Probalign and MAFFT alignments, although the last two programs would be the most likely choice for datasets of sequences with non-conserved residues at N/C terminal ends. CLUSTAL OMEGA is also indicated for alignments with non conserved terminal ends. If high performance computational resources are available, especially with multiple processing cores, recent versions of MAFFT and T-Coffee can deliver faster alignments compared to Probalign and Probcons. Parallelization of alignment is a key technique for increasing speed, which is specially suited for larger datasets than those encountered in the BAliBASE suite, and should probably be addressed by more programs in a near future.

## Competing interests

The authors declare that they have no competing interests.

## Authors’ contributions

Conceived the experiments: FSP, PCR and RSC. Performed the experiments: FSP and PCR. Analyzed the results: FSP, PCR, GO and RSC. Contributed with computational resources: GO. Wrote the paper: FSP, PCR, GO and RSC. All authors read and approved the final manuscript.

## Supplementary Material

Additional file 1 **Z-scores of SP, TC, memory and execution time measures for MSA programs in References 6, 7 and 9.** Each of the seventeen radar charts (“A” to “Q”) represents one of the BAliBASE Reference datasets 6, 7 and 9 respectively. The color lines represent the used MSA programs and dashed lines represent programs that exceed 2.5 hours of execution. The numbers represent the deviation pattern either positive, above the average, or negative, bellow the average of the programs. Click here for file

Additional file 2 **Overview of alignment accuracy from SP and TC scoring measures for BAliBASE Reference sets 1–7 and 9.** SP and TC scores of minimum, maximum, average, standard deviation and median are presented. Bold values are the highest found. The “*” represents *p* < 0.01 between compared programs. RV11: BB_SP and BB_TC: MAFFT/T-Coffee/Probalign/Probcons vs all other programs; BBS_SP and BBS_TC: Probcons/T-Coffee vs others, except MAFFT. RV12: BB_SP and BB_TC: Probalign vs others, except Probcons/T-Coffee; BBS_SP and BBS_TC: Probcons/T-Coffee vs others, except Probalign. RV20: BB_SP: MAFFT/Probalign vs others, except Probcons/T-Coffee; BBS_SP: T-Coffee/Probcons vs others, except Probalign; BB_TC: Probalign/MAFFT/Probcons vs others, except T-Coffee; BBS_TC: Probcons vs others, except T-Coffee. RV30: BB_SP: MAFFT/Probalign vs others, except T-Coffee/Probcons; BBS_SP: Probcons/T-Coffee vs others, except MAFFT/Probalign; BB_TC: MAFFT/Probalign/Probcons vs others, except T-Coffee; BBS_TC: Probcons/T-Coffee/MAFFT vs others, except Probalign. RV40: BB_SP: Probalign/MAFFT/Probcons/T-Coffee vs others; BB_TC: Probalign/MAFFT/T-Coffee vs others, except Probcons. RV50: BB_SP: Probalign/MAFFT/Probcons/T-Coffee vs others; BBS_SP: Probcons/T-Coffee vs others, except Probalign/MAFFT; BB_TC: T-Coffee vs others, except MAFFT/Probalign/Probcons; BBS_TC: Probcons/T-Coffee vs others, except MAFFT/Probalign. RV60_1a: SP: CLUSTALW/POA vs Probalign. TC: CLUSTALW vs Probalign. RV60_1b: SP: POA vs Probalign. TC: CLUSTALW/POA vs Probalign. RV60_2a: SP: POA vs Probalign/MAFFT/MUSCLE. RV60_2b: SP: CLUSTALW/DIALIGN-TX/POA vs MAFFT/Probalign/Probcons and POA vs T-Coffee. RV60_2c: SP: CLUSTALW/POA vs MAFFT/MUSCLE/Probalign/Probcons/T-Coffee and DIALIGN-TX vs MAFFT/MUSCLE/Probalign/Probcons. TC: POA vs Probalign. RV60_3: SP: CLUSTALW/POA vs Probalign/Probcons and POA vs T-Coffee. RV60_4: SP: CLUSTALW/POA/DIALIGN-TX/MUSCLE vs Probalign. RV70: SP: DIALIGN-TX vs Probcons and POA vs MAFFT/Probcons/T-Coffee. TC: POA vs MAFFT/Probcons. RV911: SP: CLUSTALW/POA/CLUSTAL OMEGA vs Probcons/T-Coffee. RV912: SP: CLUSTAL OMEGA vs Probalign/Probcons/T-Coffee. TC: CLUSTAL OMEGA vs Probalign/Probcons/T-Coffee. RV913: SP: CLUSTAL OMEGA vs CLUSTALW/MAFFT/MUSCLE/Probalign/Probcons/T-Coffee. TC: CLUSTAL OMEGA vs Probalign/Probcons/T-Coffee. RV931: SP: POA vs Probcons/T-Coffee. RV941: SP: CLUSTALW/POA vs Probalign/Probcons/T-Coffee, Dialing vs Probcons and MAFFT vs POA. TC: POA vs Probcons. RV942: SP: CLUSTALW/POA/CLUSTAL OMEGA vs Probcons/T-Coffee and DIALIGN-TX vs T-Coffee. Click here for file

Additional file 3 **Overview of computational cost from execution time measure for BAliBASE Reference sets 1–7 and 9.** Execution times are presented for each program, reference set and sequence type (full-length or truncated), when available. Values in bold are the smallest found. Execution times over 2.5 hours were not applicable (N/A) in this study. Click here for file

Additional file 4 **Overview of computational costs from memory usage measure from BAliBASE Reference sets 1–7 and 9.** Memory usage (kb) are presented for each program, reference set and sequence type (full-length or truncated), when available. Values in bold are the smallest found. For programs where execution times exceeded 2.5 hours, memory usage was not applicable (N/A) in this study. Click here for file
