# Supplementary figures and images for: Assessing the efficiency of multiple sequence alignment programs
Source: Algorithms Mol Biol. 2014 Mar 6;9:4. doi: 10.1186/1748-7188-9-4 (PMC4015676; doi:10.1186/1748-7188-9-4)

A)

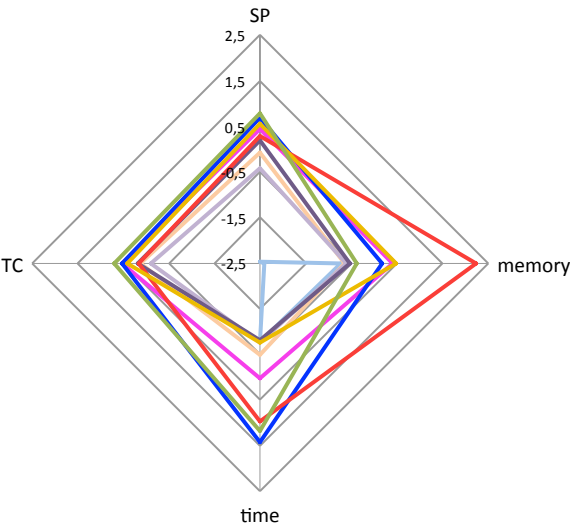

B)

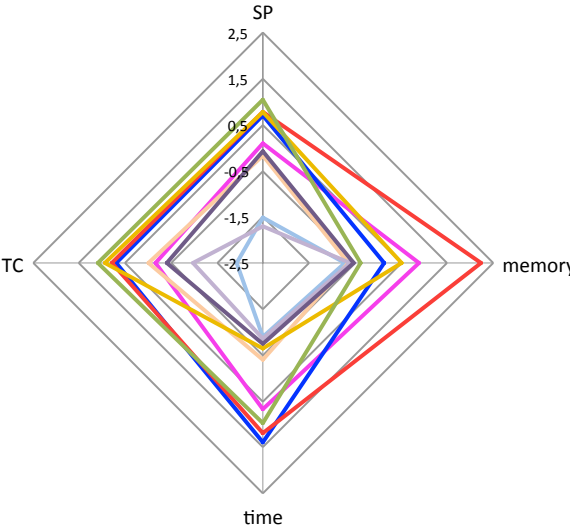

C)

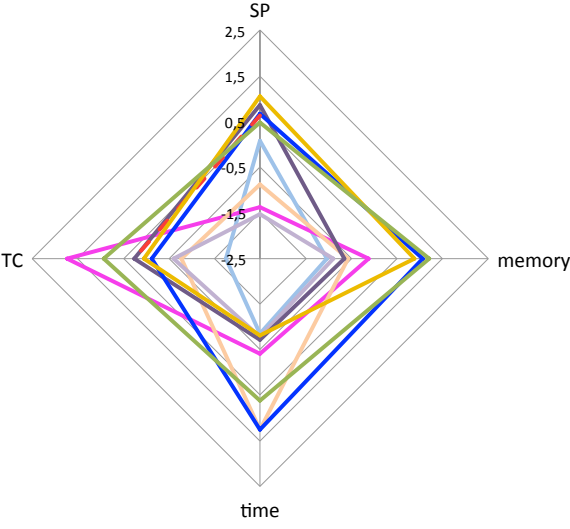

D)

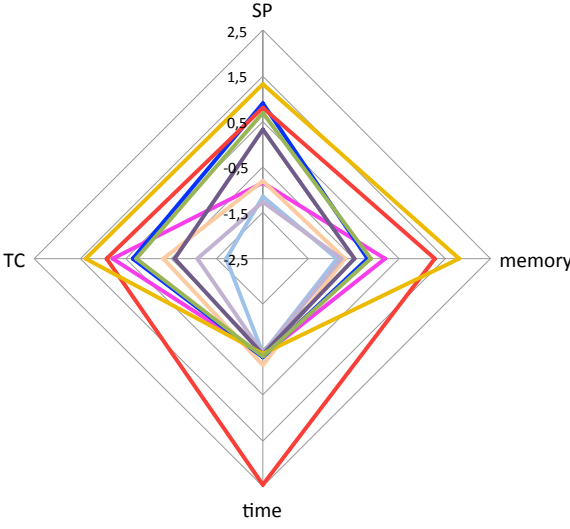

E)

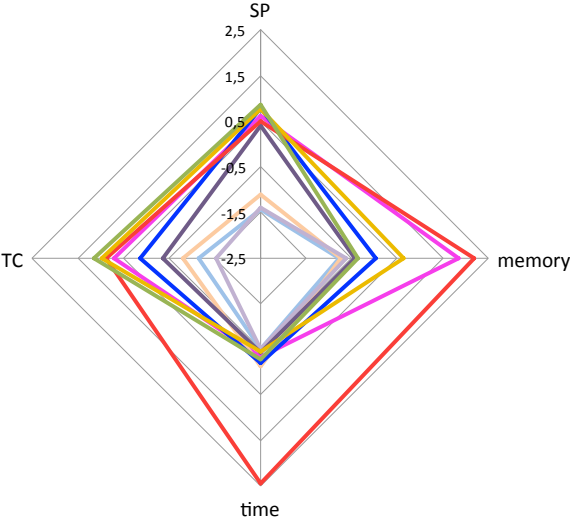

F)

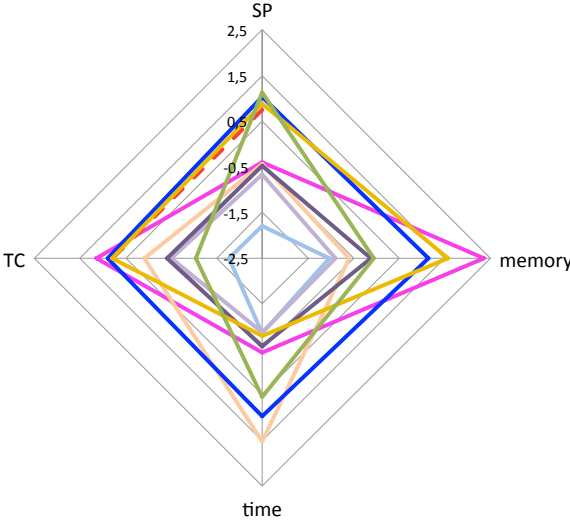

G)

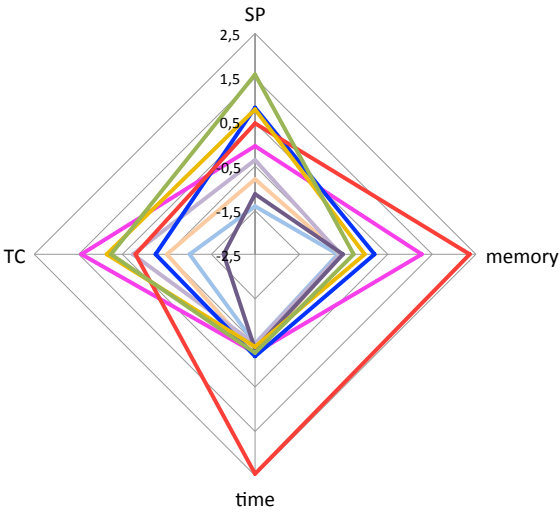

H)

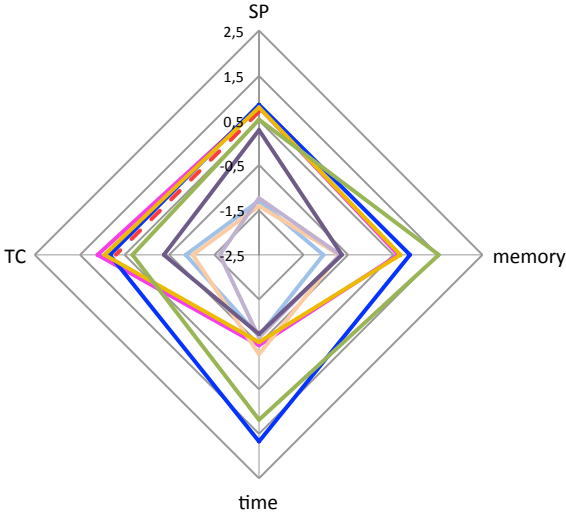

I)

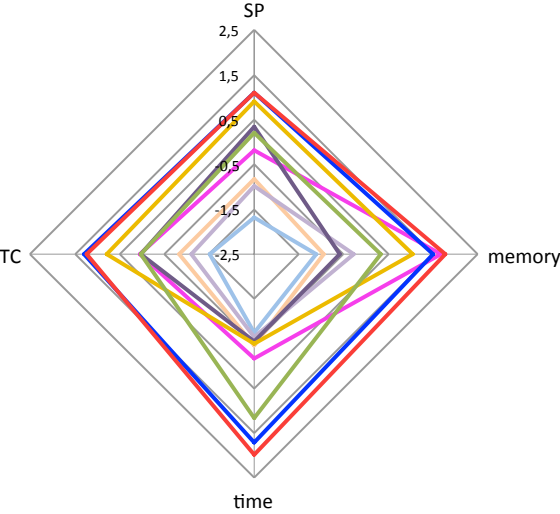

J)

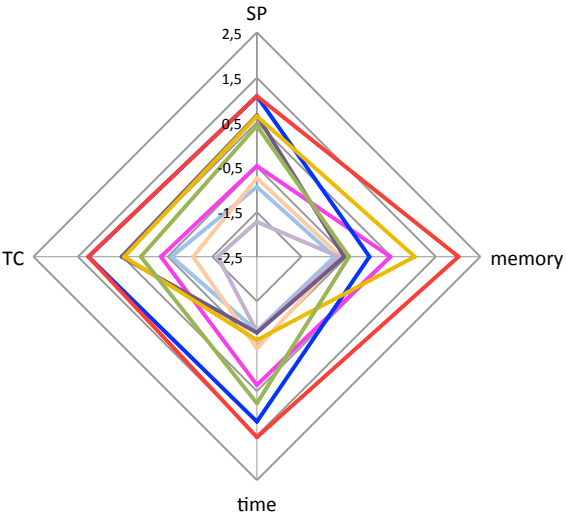

K)

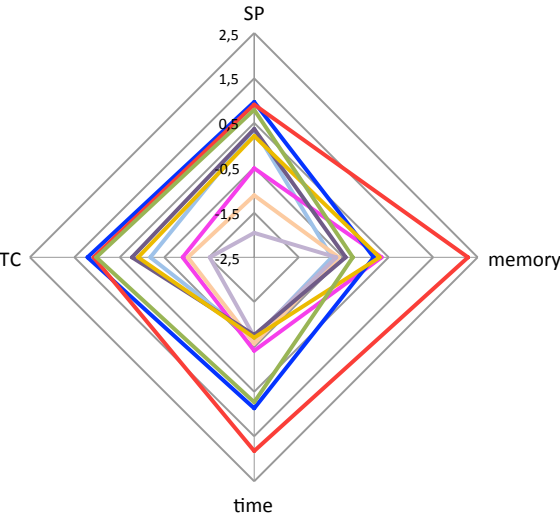

L)

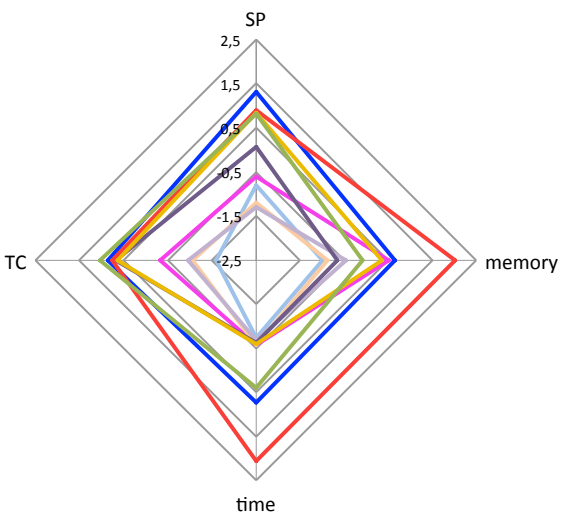

M)

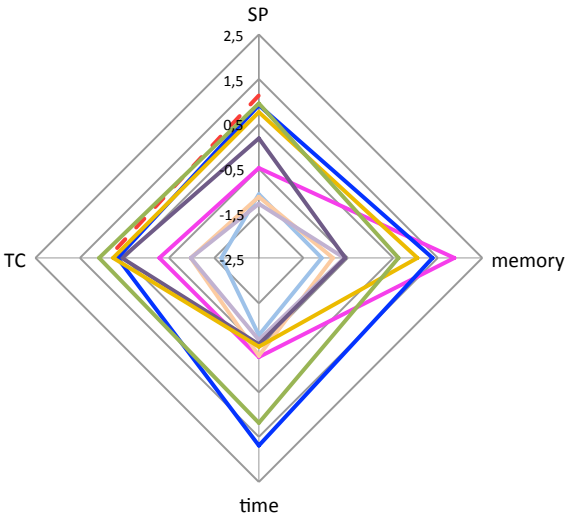

N)

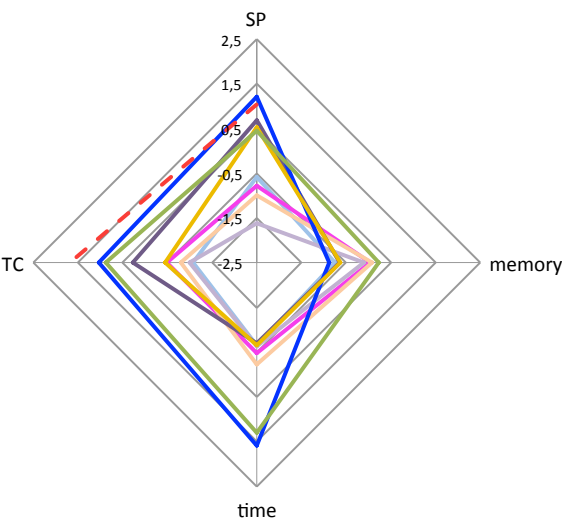

O)

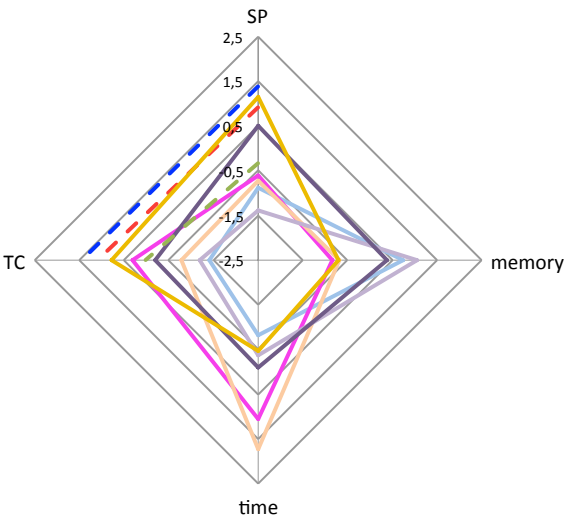

P)

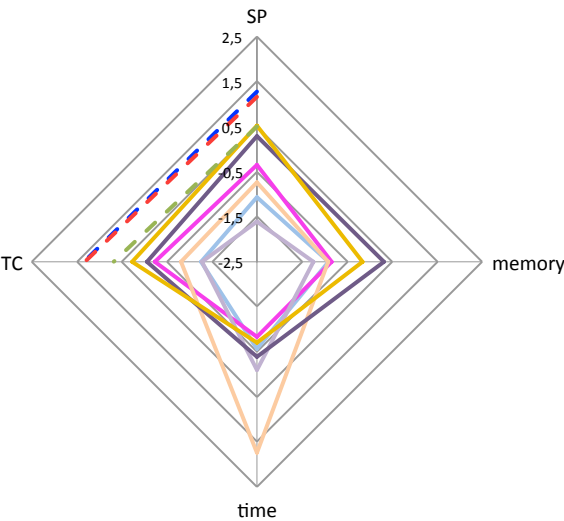

Q)

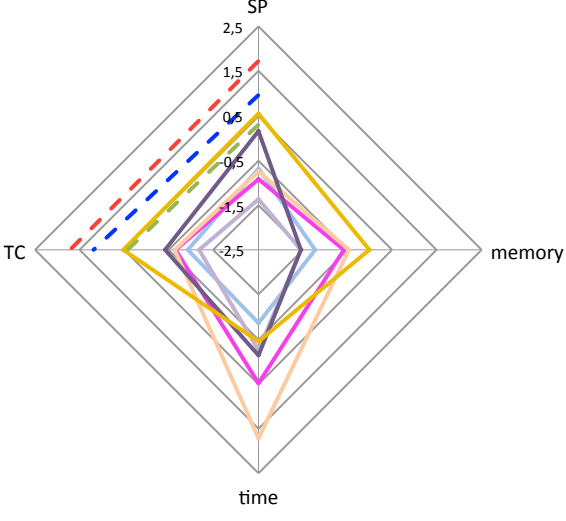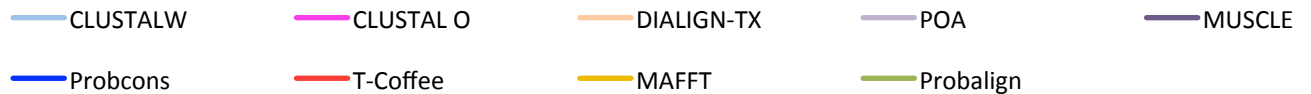

Supplement: Additional file 1 — Z-scores of SP, TC, memory and execution time measures for MSA programs in References 6, 7 and 9. Each of the seventeen radar charts (“A” to “Q”) represents one of the BAliBASE Reference datasets 6, 7 and 9 respectively. The color lines represent the used MSA programs and dashed lines represent programs that exceed 2.5 hours of execution. The numbers represent the deviation pattern either positive, above the average, or negative, bellow the average of the programs. [file 1748-7188-9-4-S1.pdf]
