# Supplementary material for: Assessing the efficiency of multiple sequence alignment programs
Source: Algorithms Mol Biol. 2014 Mar 6;9:4. doi: 10.1186/1748-7188-9-4 (PMC4015676; doi:10.1186/1748-7188-9-4)
Supplement: Additional file 2 — Overview of alignment accuracy from SP and TC scoring measures for BAliBASE Reference sets 1–7 and 9. SP and TC scores of minimum, maximum, average, standard deviation and median are presented. Bold values are the highest found. The “*” represents p < 0.01 between compared programs. RV11: BB_SP and BB_TC: MAFFT/T-Coffee/Probalign/Probcons vs all other programs; BBS_SP and BBS_TC: Probcons/T-Coffee vs others, except MAFFT. RV12: BB_SP and BB_TC: Probalign vs others, except Probcons/T-Coffee; BBS_SP and BBS_TC: Probcons/T-Coffee vs others, except Probalign. RV20: BB_SP: MAFFT/Probalign vs others, except Probcons/T-Coffee; BBS_SP: T-Coffee/Probcons vs others, except Probalign; BB_TC: Probalign/MAFFT/Probcons vs others, except T-Coffee; BBS_TC: Probcons vs others, except T-Coffee. RV30: BB_SP: MAFFT/Probalign vs others, except T-Coffee/Probcons; BBS_SP: Probcons/T-Coffee vs others, except MAFFT/Probalign; BB_TC: MAFFT/Probalign/Probcons vs others, except T-Coffee; BBS_TC: Probcons/T-Coffee/MAFFT vs others, except Probalign. RV40: BB_SP: Probalign/MAFFT/Probcons/T-Coffee vs others; BB_TC: Probalign/MAFFT/T-Coffee vs others, except Probcons. RV50: BB_SP: Probalign/MAFFT/Probcons/T-Coffee vs others; BBS_SP: Probcons/T-Coffee vs others, except Probalign/MAFFT; BB_TC: T-Coffee vs others, except MAFFT/Probalign/Probcons; BBS_TC: Probcons/T-Coffee vs others, except MAFFT/Probalign. RV60_1a: SP: CLUSTALW/POA vs Probalign. TC: CLUSTALW vs Probalign. RV60_1b: SP: POA vs Probalign. TC: CLUSTALW/POA vs Probalign. RV60_2a: SP: POA vs Probalign/MAFFT/MUSCLE. RV60_2b: SP: CLUSTALW/DIALIGN-TX/POA vs MAFFT/Probalign/Probcons and POA vs T-Coffee. RV60_2c: SP: CLUSTALW/POA vs MAFFT/MUSCLE/Probalign/Probcons/T-Coffee and DIALIGN-TX vs MAFFT/MUSCLE/Probalign/Probcons. TC: POA vs Probalign. RV60_3: SP: CLUSTALW/POA vs Probalign/Probcons and POA vs T-Coffee. RV60_4: SP: CLUSTALW/POA/DIALIGN-TX/MUSCLE vs Probalign. RV70: SP: DIALIGN-TX vs Probcons and POA vs MAFFT/Probcons/T-Coffee. TC: POA vs [file 1748-7188-9-4-S2.pdf]

| | | Minimum | Maximum | Average | Standard Deviation | Median | Minimum | Maximum | Average | Standard Deviation | Median | Minimum | Maximum | Average | Standard Deviation | Median | Minimum | Maximum | Average | Standard Deviation | Median |
|---|---|---|---|---|---|---|---|---|---|---|---|---|---|---|---|---|---|---|---|---|---|
| | | **BB_SP*** | | | | | **BBS_SP*** | | | | | **BB_TC*** | | | | | **BBS_TC*** | | | | |
| **RV11** | **CLUSTALW** | 0.084 | **0.961** | 0.415 | 0.203 | 0.406 | 0.275 | 0.974 | 0.566 | 0.176 | 0.542 | 0.000 | **0.920** | 0.170 | 0.239 | 0.080 | 0.000 | 0.940 | 0.308 | 0.237 | 0.235 |
| | **CLUSTAL O.** | 0.063 | 0,908 | 0,491 | 0.208 | 0.518 | 0.041 | 0.947 | 0.544 | 0.194 | 0.553 | 0.000 | 0.810 | 0.255 | 0.226 | 0.230 | 0.000 | 0.890 | 0.301 | 0.234 | 0.255 |
| | **DIALIGN-TX** | 0.014 | 0.895 | 0.416 | 0.202 | 0.406 | 0.076 | 0.906 | 0.496 | 0.197 | 0.503 | 0.000 | 0.820 | 0.185 | 0.215 | 0.090 | 0.000 | 0.840 | 0.257 | 0.216 | 0.245 |
| | **MAFFT** | 0.042 | 0.917 | 0.538 | 0.200 | 0.571 | 0.178 | 0.980 | 0.623 | 0.164 | 0.645 | 0.000 | 0.840 | 0.297 | 0.219 | 0.270 | 0.000 | 0.960 | 0.392 | 0.214 | 0.340 |
| | **MUSCLE** | 0.073 | 0.904 | 0.465 | 0.211 | 0.426 | 0.380 | **0.993** | 0.630 | 0.147 | 0.567 | 0.000 | 0.850 | 0.228 | 0.245 | 0.150 | 0.000 | **0.980** | 0.396 | 0.230 | 0.390 |
| | **POA** | 0.000 | 0.855 | 0.349 | 0.186 | 0.300 | 0.000 | 0.856 | 0.373 | 0.182 | 0.365 | 0.000 | 0.760 | 0.132 | 0.177 | 0.095 | 0.000 | 0.760 | 0.143 | 0.180 | 0.115 |
| | **Probalign** | 0.084 | 0.927 | **0.571** | 0.195 | **0.582** | 0.078 | 0.932 | 0.621 | 0.179 | 0.639 | 0.000 | 0.880 | 0.318 | 0.243 | **0.275** | 0.000 | 0.890 | 0.371 | 0.240 | 0.325 |
| | **Probcons** | **0.137** | 0.939 | 0.547 | 0.191 | 0.541 | **0.414** | **0.993** | **0.705** | 0.128 | 0.705 | 0.000 | 0.890 | 0.296 | 0.243 | 0.265 | **0.190** | **0.980** | **0.485** | 0.207 | **0.490** |
| | **T-Coffee** | 0.105 | 0.921 | 0.549 | 0.194 | 0.546 | 0.401 | **0.993** | 0.699 | 0.128 | **0.711** | 0.000 | 0.860 | **0.299** | 0.241 | 0.245 | 0.180 | **0.980** | 0.482 | **0.205** | 0.465 |
| | | **BB_SP*** | | | | | **BBS_SP*** | | | | | **BB_TC*** | | | | | **BBS_TC*** | | | | |
| **RV12** | **CLUSTALW** | 0.317 | **1,000** | 0.799 | 0.130 | 0.836 | 0.535 | **1,000** | 0.839 | 0.100 | 0.867 | 0.000 | **1,000** | 0.605 | 0.210 | 0.595 | 0.370 | **1,000** | 0.678 | 0.161 | 0.690 |
| | **CLUSTAL O.** | 0.304 | **1.000** | 0.853 | 0.124 | 0.893 | 0.304 | **1.000** | 0.856 | 0.123 | 0.894 | 0.000 | **1.000** | 0.696 | 0.188 | 0.720 | 0.000 | **1.000** | 0.701 | 0.184 | 0.740 |
| | **DIALIGN-TX** | 0.558 | 0.959 | 0.819 | 0.096 | 0.847 | 0.566 | 0.951 | 0.828 | 0.095 | 0.850 | 0.340 | 0.900 | 0.631 | 0.153 | 0.625 | 0.330 | 0.920 | 0.636 | 0.155 | 0.650 |
| | **MAFFT** | 0.572 | 0.986 | 0.863 | 0.080 | 0.889 | 0.587 | 0.995 | 0.877 | 0.077 | 0.897 | 0.390 | 0.930 | 0.705 | 0.126 | 0.710 | 0.370 | 0.970 | 0.725 | 0.128 | 0.735 |
| | **MUSCLE** | 0.550 | 0.964 | 0.845 | 0.089 | 0.868 | 0.603 | **1,000** | 0.869 | 0.081 | 0.894 | 0.390 | 0.890 | 0.679 | 0.142 | 0.695 | 0.410 | **1,000** | 0.716 | 0.139 | 0.725 |
| | **POA** | 0.272 | 0.959 | 0.778 | 0.128 | 0.807 | 0.272 | 0.917 | 0.777 | 0.128 | 0.814 | 0.000 | 0.830 | 0.567 | 0.176 | 0.615 | 0.000 | 0.830 | 0.565 | 0.172 | 0.615 |
| | **Probalign** | **0.603** | **1,000** | **0.883** | 0.073 | **0.895** | 0.603 | **1,000** | 0.888 | 0.074 | 0.901 | **0.470** | **1,000** | **0.740** | 0.124 | **0.750** | **0.450** | **1,000** | 0.748 | 0.123 | 0.765 |
| | **Probcons** | 0.568 | 0.995 | 0.875 | 0.081 | 0.888 | 0.599 | **1,000** | 0.894 | 0.074 | **0.909** | 0.430 | 0.970 | 0.734 | 0.131 | 0.730 | 0.440 | **1,000** | **0.761** | 0.125 | **0.775** |
| | **T-Coffee** | 0.577 | 0.995 | 0.874 | 0.077 | 0.888 | **0.613** | **1,000** | **0.896** | 0.068 | 0.905 | 0.410 | 0.970 | 0.729 | 0.129 | 0.730 | **0.450** | **1,000** | 0.757 | **0.123** | 0.750 |
| | | **BB_SP*** | | | | | **BBS_SP*** | | | | | **BB_TC*** | | | | | **BBS_TC*** | | | | |
| **RV20** | **CLUSTALW** | 0.164 | 0.955 | 0.769 | 0.168 | 0.805 | 0.747 | **0.987** | 0.874 | 0.069 | 0.892 | 0.000 | 0.630 | 0.182 | 0.207 | 0.080 | 0.010 | 0.900 | 0.338 | 0.201 | 0.310 |
| | **CLUSTAL O.** | 0.451 | 0.977 | 0.834 | 0.124 | 0.866 | 0.755 | 0.978 | 0.898 | 0.056 | 0.907 | 0.000 | **0.720** | 0.315 | 0.216 | **0.300** | 0.050 | 0.720 | 0.372 | 0.210 | 0.380 |
| | **DIALIGN-TX** | 0.486 | 0.959 | 0.799 | 0.119 | 0.810 | 0.704 | 0.972 | 0.860 | 0.068 | 0.862 | 0.000 | 0.650 | 0.227 | 0.178 | 0.210 | 0.020 | 0.670 | 0.297 | 0.186 | 0.290 |
| | **MAFFT** | 0.519 | 0.975 | **0.846** | 0.110 | **0.881** | **0.790** | 0.980 | 0.907 | 0.049 | 0.911 | 0.000 | 0.700 | **0.309** | 0.207 | 0.290 | 0.050 | 0.930 | 0.386 | 0.206 | 0.370 |
| | **MUSCLE** | 0.217 | 0.972 | 0.820 | 0.154 | 0.844 | 0.778 | 0.976 | 0.899 | 0.053 | 0.895 | 0.000 | 0.680 | 0.248 | 0.214 | 0.210 | 0.040 | 0.900 | 0.357 | 0.198 | 0.340 |
| | **POA** | 0.272 | 0.947 | 0.777 | 0.150 | 0.809 | 0.692 | 0.956 | 0.847 | 0.075 | 0.833 | 0.000 | 0.610 | 0.206 | 0.201 | 0.150 | 0.020 | 0.790 | 0.270 | 0.198 | 0.230 |
| | **Probalign** | 0.490 | **0.982** | **0.846** | 0.113 | 0.880 | 0.789 | 0.983 | 0.907 | 0.050 | 0.913 | 0.000 | **0.720** | 0.307 | 0.224 | 0.290 | 0.010 | 0.940 | 0.369 | 0.230 | 0.290 |
| | **Probcons** | 0.477 | 0.978 | 0.834 | 0.114 | 0.864 | **0.790** | 0.985 | **0.920** | 0.045 | **0.926** | 0.000 | 0.700 | 0.291 | 0.220 | 0.280 | **0.120** | **0.950** | **0.465** | 0.194 | **0.440** |
| | **T-Coffee** | **0.527** | 0 .976 | 0.833 | 0.114 | 0.859 | **0.790** | 0.984 | 0.918 | 0.046 | **0.926** | 0.000 | 0.700 | 0.279 | 0.220 | 0.270 | 0.110 | 0.940 | 0.443 | **0.194** | 0.420 |
| | | **BB_SP*** | | | | | **BBS_SP*** | | | | | **BB_TC*** | | | | | **BBS_TC*** | | | | |
| **RV30** | **CLUSTALW** | 0.396 | 0.925 | 0.638 | 0.143 | 0.599 | 0.470 | 0.905 | 0.729 | 0.119 | 0.744 | 0.000 | 0.650 | 0.169 | 0.173 | 0.130 | 0.000 | 0.640 | 0.326 | 0.175 | 0.290 |
| | **CLUSTAL O.** | **0.490** | **0.953** | 0.763 | 0.117 | 0.768 | 0.524 | **0.956** | 0.777 | 0.118 | 0.779 | 0.000 | **0.770** | **0.384** | 0.186 | 0.380 | 0.000 | **0.760** | 0.420 | 0.175 | 0.400 |
| | **DIALIGN-TX** | 0.348 | 0.890 | 0.674 | 0.134 | 0.689 | 0.504 | 0.908 | 0.699 | 0.119 | 0.701 | 0.000 | 0.570 | 0.237 | 0.155 | 0.245 | 0.000 | 0.540 | 0.276 | 0.149 | 0.270 |
| | **MAFFT** | 0.427 | 0.948 | **0.769** | 0.129 | **0.799** | 0.542 | 0.948 | 0.803 | 0.105 | 0.831 | 0.000 | 0.750 | 0.383 | 0.195 | **0.400** | 0.060 | **0.760** | 0.442 | 0.177 | 0.435 |
| | **MUSCLE** | 0.375 | 0.921 | 0.717 | 0.132 | 0.731 | 0.555 | 0.936 | 0.783 | 0.107 | 0.788 | 0.000 | 0.650 | 0.246 | 0.184 | 0.250 | 0.050 | 0.690 | 0.372 | 0.169 | 0.355 |
| | **POA** | 0.423 | 0.886 | 0.652 | 0.139 | 0.649 | 0.446 | 0.922 | 0.681 | 0.138 | 0.667 | 0.000 | 0.620 | 0.201 | 0.168 | 0.160 | 0.000 | 0.630 | 0.257 | 0.180 | 0.185 |
| | **Probalign** | 0.455 | 0.948 | 0.756 | 0.126 | 0.764 | 0.507 | 0.947 | 0.788 | 0.110 | 0.796 | 0.000 | 0.720 | 0.362 | 0.180 | 0.380 | 0.000 | 0.750 | 0.415 | 0.176 | 0.390 |
| | **Probcons** | 0.399 | 0.943 | 0.751 | 0.132 | 0.769 | 0.637 | 0.953 | 0.824 | 0.085 | 0.834 | 0.000 | 0.730 | 0.343 | 0.181 | 0.350 | 0.110 | **0.760** | 0.463 | 0.152 | **0.455** |
| | **T-Coffee** | 0.384 | 0.941 | 0.739 | 0.135 | 0.762 | **0.653** | 0.951 | **0.827** | 0.078 | **0.838** | 0.000 | 0.710 | 0.319 | 0.187 | 0.340 | **0.160** | 0.740 | **0.466** | **0.151** | **0.455** |
| | | **BB_SP*** | | | | | **BBS_SP** | | | | | **BB_TC*** | | | | | **BBS_TC** | | | | |
| **RV40** | **CLUSTALW** | 0.221 | 0.920 | 0.695 | 0.180 | 0.746 | - | - | - | - | - | 0.000 | 0.980 | 0.302 | 0.285 | 0.330 | - | - | - | - | - |
| | **CLUSTAL O.** | 0.518 | **0.980** | 0.813 | 0.105 | 0.850 | - | - | - | - | - | 0.000 | **0.990** | 0.446 | 0.281 | **0.510** | - | - | - | - | - |
| | **DIALIGN-TX** | 0.369 | 0.944 | 0.739 | 0.130 | 0.773 | - | - | - | - | - | 0.000 | 0.960 | 0.331 | 0.264 | 0.340 | - | - | - | - | - |
| | **MAFFT** | **0.585** | 0.947 | 0.812 | 0.089 | 0.843 | - | - | - | - | - | 0.000 | 0.970 | 0.441 | 0.267 | 0.470 | - | - | - | - | - |
| | **MUSCLE** | 0.416 | 0.933 | 0.759 | 0.143 | 0.820 | - | - | - | - | - | 0.000 | 0.960 | 0.338 | 0.289 | 0.380 | - | - | - | - | - |
| | **POA** | 0.379 | 0.897 | 0.710 | 0.145 | 0.758 | - | - | - | - | - | 0.000 | 0.900 | 0.315 | 0.246 | 0.320 | - | - | - | - | - |
| | **Probalign** | 0.462 | 0.944 | **0.823** | 0.104 | **0.865** | - | - | - | - | - | 0.000 | **0.990** | **0.452** | 0.278 | 0.490 | - | - | - | - | - |
| | **Probcons** | 0.498 | 0.941 | 0.805 | 0.108 | 0.839 | - | - | - | - | - | 0.000 | 0.980 | 0.404 | 0.282 | 0.420 | - | - | - | - | - |
| | **T-Coffee** | 0.423 | 0.940 | 0.800 | 0.116 | 0.838 | - | - | - | - | - | 0.000 | 0.960 | 0.419 | 0.270 | 0.430 | - | - | - | - | - |
| | | **BB_SP*** | | | | | **BBS_SP*** | | | | | **BB_TC*** | | | | | **BBS_TC*** | | | | |
| **RV50** | **CLUSTALW** | 0.237 | 0.945 | 0.637 | 0.200 | 0.615 | 0.326 | 0.921 | 0.704 | 0.154 | 0.704 | 0.000 | 0.810 | 0.236 | 0.307 | 0.045 | 0.000 | 0.730 | 0.296 | 0.244 | 0.190 |
| | **CLUSTAL O.** | **0.473** | 0.949 | 0.752 | 0.138 | 0.734 | 0.455 | 0.945 | 0.765 | 0.135 | 0.791 | 0.000 | 0.850 | 0.373 | 0.265 | 0.310 | 0.000 | 0.740 | 0.364 | 0.247 | 0.290 |
| | **DIALIGN-TX** | 0.362 | 0.933 | 0.692 | 0.153 | 0.693 | 0.442 | 0.903 | 0.717 | 0.127 | 0.755 | 0.000 | 0.790 | 0.316 | 0.231 | 0.240 | 0.000 | 0.650 | 0.305 | 0.196 | 0.250 |
| | **MAFFT** | 0.458 | **0.962** | **0.774** | 0.130 | 0.774 | 0.525 | 0.934 | 0.803 | 0.110 | 0.834 | 0.000 | **0.880** | 0.393 | 0.258 | **0.360** | 0.050 | 0.730 | 0.406 | 0.247 | 0.370 |
| | **MUSCLE** | 0.270 | 0.949 | 0.702 | 0.170 | 0.707 | 0.459 | 0.928 | 0.763 | 0.121 | 0.781 | 0.000 | 0.840 | 0.304 | 0.285 | 0.220 | 0.000 | 0.720 | 0.346 | 0.234 | 0.380 |
| | **POA** | 0.357 | 0.925 | 0.655 | 0.153 | 0.613 | 0.343 | 0.884 | 0.671 | 0.138 | 0.683 | 0.000 | 0.800 | 0.241 | 0.245 | 0.120 | 0.040 | 0.640 | 0.242 | 0.194 | 0.150 |
| | **Probalign** | 0.454 | 0.961 | 0.766 | 0.135 | **0.776** | 0.522 | 0.939 | 0.797 | 0.115 | 0.829 | **0.020** | **0.880** | 0.375 | 0.267 | 0.350 | 0.010 | 0.740 | 0.373 | 0.251 | 0.380 |
| | **Probcons** | 0.407 | 0.956 | 0.766 | 0.141 | 0.770 | 0.540 | **0.954** | **0.814** | 0.110 | **0.846** | 0.000 | 0.860 | 0.386 | 0.261 | 0.330 | **0.100** | **0.800** | 0.444 | 0.235 | 0.410 |
| | **T-Coffee** | 0.396 | 0.961 | 0.763 | 0.143 | 0.773 | **0.568** | 0.952 | 0.811 | 0.108 | 0.842 | 0.000 | **0.880** | **0.396** | 0.262 | 0.350 | **0.100** | **0.800** | **0.450** | 0.229 | **0.430** |

| | | Minimum | Maximum | Average | Standard Deviation | Median | Minimum | Maximum | Average | Standard Deviation | Median |
|---|---|---|---|---|---|---|---|---|---|---|---|
| | | SP* | | | | | TC* | | | | |
| RV60_1a | CLUSTALW | 0.473 | 0.849 | 0.669 | 0.153 | 0.677 | 0.000 | 0.670 | 0.167 | 0.335 | 0.000 |
| | CLUSTAL O. | 0.755 | **0.974** | 0.889 | 0.103 | 0.914 | 0.500 | **0.910** | 0.750 | 0.189 | **0.795** |
| | DIALIGN-TX | 0.718 | 0.931 | 0.849 | 0.093 | 0.873 | 0.430 | 0.840 | 0.675 | 0.177 | 0.715 |
| | MAFFT | 0.847 | 0.945 | 0.897 | 0.052 | 0.899 | 0.580 | 0.840 | 0.727 | 0.114 | 0.745 |
| | MUSCLE | 0.834 | 0.918 | 0.869 | 0.036 | 0.863 | 0.600 | 0.760 | 0.685 | 0.065 | 0.690 |
| | POA | 0.702 | 0.897 | 0.823 | 0.085 | 0.848 | 0.430 | 0.760 | 0.630 | 0.142 | 0.665 |
| | Probalign | **0.862** | 0.970 | **0.915** | 0.050 | 0.914 | **0.690** | 0.880 | **0.780** | 0.094 | 0.775 |
| | Probcons | 0.860 | 0.929 | 0.907 | 0.031 | **0.920** | **0.690** | 0.840 | 0.750 | 0.066 | 0.735 |
| | T-Coffee | 0.812 | 0.931 | 0.877 | 0.053 | 0.883 | 0.500 | 0.840 | 0.682 | 0.139 | 0.695 |
| | | SP* | | | | | TC* | | | | |
| RV60_1b | CLUSTALW | 0.620 | 0.919 | 0.762 | 0.115 | 0.776 | 0.000 | 0.700 | 0.248 | 0.344 | 0.000 |
| | CLUSTAL O. | 0.729 | **0.990** | 0.863 | 0.111 | 0.886 | 0.000 | **0.980** | 0.572 | 0.372 | 0.540 |
| | DIALIGN-TX | 0.733 | 0.926 | 0.846 | 0.074 | 0.859 | 0.420 | 0.750 | 0.602 | 0.155 | 0.670 |
| | MAFFT | 0.775 | 0.963 | 0.906 | 0.076 | 0.929 | 0.550 | 0.920 | 0.774 | 0.147 | 0.820 |
| | MUSCLE | 0.730 | 0.930 | 0.852 | 0.089 | 0.906 | 0.000 | 0.800 | 0.526 | 0.331 | 0.710 |
| | POA | 0.685 | 0.870 | 0.750 | 0.073 | 0.735 | 0.000 | 0.770 | 0.422 | 0.279 | 0.410 |
| | Probalign | 0.777 | 0.979 | **0.922** | 0.086 | **0.972** | 0.550 | 0.960 | **0.802** | 0.179 | **0.850** |
| | Probcons | **0.779** | 0.976 | 0.901 | 0.074 | 0.914 | **0.570** | 0.940 | 0.732 | 0.158 | 0.750 |
| | T-Coffee | 0.775 | 0.969 | 0.906 | 0.079 | 0.919 | 0.550 | 0.900 | 0.748 | 0.174 | 0.820 |
| | | SP* | | | | | TC* | | | | |
| RV60_2a | CLUSTALW | 0.324 | 0.971 | 0.812 | 0.189 | 0.854 | 0.000 | 0.910 | 0.273 | 0.364 | 0.000 |
| | CLUSTAL O. | 0.313 | 0.990 | 0.694 | 0.292 | 0.720 | 0.000 | **0.960** | **0.432** | 0.442 | **0.370** |
| | DIALIGN-TX | 0.223 | 0.990 | 0.735 | 0.247 | 0.849 | 0.000 | **0.960** | 0.317 | 0.415 | 0.000 |
| | MAFFT | **0.742** | 0.991 | **0.891** | 0.102 | **0.956** | 0.000 | **0.960** | 0.355 | 0.403 | 195 |
| | MUSCLE | 0.510 | 0.979 | 0.876 | 0.134 | 0.941 | 0.000 | 0.930 | 0.365 | 0.404 | 0.215 |
| | POA | 0.242 | 0.967 | 0.682 | 0.261 | 0.675 | 0.000 | 0.920 | 0.325 | 0.378 | 0.165 |
| | Probalign | 0.301 | **0.998** | 0.845 | 0.208 | 0.938 | 0.000 | 0.950 | 0.395 | 0.430 | 0.250 |
| | Probcons | 0.680 | 0.985 | 0.860 | 0.108 | 0.877 | 0.000 | 0.950 | 0.347 | 0.416 | 0.065 |
| | T-Coffee | 0.616 | 0.981 | 0.857 | 0.112 | 0.887 | 0.000 | 0.950 | 0.365 | 0.407 | 0.185 |
| | | SP* | | | | | TC | | | | |
| RV60_2b | CLUSTALW | 0.345 | 0.938 | 0.616 | 0.198 | 0.581 | 0.000 | 0.790 | 0.115 | 0.264 | 0.000 |
| | CLUSTAL O. | 0.182 | 0.980 | 0.642 | 0.293 | 0.700 | 0.000 | 0.900 | 0.285 | 0.380 | 0.000 |
| | DIALIGN-TX | 0.259 | 0.964 | 0.645 | 0.213 | 0.575 | 0.000 | 0.890 | 0.209 | 0.347 | 0.000 |
| | MAFFT | **0.624** | **0.987** | **0.821** | 0.124 | **0.865** | 0.000 | **0.940** | **0.325** | 0.339 | **0.360** |
| | MUSCLE | 0.364 | 0.972 | 0.739 | 0.170 | 0.743 | 0.000 | 0.820 | 0.192 | 0.302 | 0.000 |
| | POA | 0.209 | 0.942 | 0.607 | 0.221 | 0.620 | 0.000 | 0.840 | 0.157 | 0.302 | 0.000 |
| | Probalign | 0.366 | 0.975 | 0.768 | 0.187 | 0.783 | 0.000 | 0.890 | 0.249 | 0.349 | 0.000 |
| | Probcons | 0.354 | 0.977 | 0.786 | 0.158 | 0.836 | 0.000 | **0.940** | 0.254 | 0.381 | 0.000 |
| | T-Coffee | 0.376 | 0.971 | 0.778 | 0.154 | 0.851 | 0.000 | 0.890 | 0.294 | 0.349 | 0.150 |
| | | SP* | | | | | TC* | | | | |
| RV60_2c | CLUSTALW | 0.186 | 0.963 | 0.669 | 0.215 | 0.705 | 0.000 | 0.770 | 0.200 | 0.295 | 0.000 |
| | CLUSTAL O. | 0.325 | 0.980 | 0.796 | 0.164 | 0.839 | 0.000 | 0.820 | 0.324 | 0.344 | 0.230 |
| | DIALIGN-TX | 0.280 | 0.984 | 0.689 | 0.190 | 0.729 | 0.000 | 0.840 | 0.222 | 0.293 | 0.000 |
| | MAFFT | **0.579** | 0.983 | 0.806 | 0.122 | 0.803 | 0.000 | **0.850** | 0.342 | 0.333 | 0.305 |
| | MUSCLE | 0.442 | 0.982 | 0.783 | 0.138 | 0.815 | 0.000 | 0.810 | 0.252 | 0.320 | 0.000 |
| | POA | 0.291 | 0.982 | 0.672 | 0.189 | 0.717 | 0.000 | 0.740 | 0.174 | 0.276 | 0.000 |
| | Probalign | 0.445 | **0.985** | **0.812** | 0.146 | **0.853** | 0.000 | 0.810 | **0.354** | 0.340 | 0.340 |
| | Probcons | 0.490 | **0.985** | 0.807 | 0.121 | 0.835 | 0.000 | 0.790 | 0.286 | 0.329 | 0.90 |
| | T-Coffee | 0.483 | 0.979 | 0.789 | 0.137 | 0.814 | 0.000 | 0.780 | 0.335 | 0.335 | **0.360** |
| | | SP* | | | | | TC | | | | |
| RV60_3 | CLUSTALW | 0.259 | 0.967 | 0.761 | 0.235 | 0.855 | 0.000 | 0.830 | 0.323 | 0.397 | 0.000 |
| | CLUSTAL O. | 0.435 | **0.980** | 0.819 | 0.215 | 0.921 | 0.000 | 0.890 | **0.485** | 0.412 | **0.660** |
| | DIALIGN-TX | 0.613 | 0.965 | 0.818 | 0.147 | 0.887 | 0.000 | 0.820 | 0.426 | 0.342 | 0.540 |
| | MAFFT | **0.670** | 0.974 | 0.873 | 0.102 | 0.891 | 0.000 | 0.850 | 0.466 | 0.374 | 0.500 |
| | MUSCLE | 0.290 | 0.977 | 0.816 | 0.221 | 0.910 | 0.000 | 0.890 | 0.399 | 0.395 | 0.540 |
| | POA | 0.584 | 0.965 | 0.808 | 0.133 | 0.837 | 0.000 | 0.810 | 0.394 | 0.331 | 0.510 |
| | Probalign | 0.611 | 0.971 | **0.883** | 0.121 | **0.927** | 0.000 | **0.910** | 0.364 | 0.423 | 0.060 |
| | Probcons | 0.646 | 0.977 | 0.879 | 0.111 | 0.904 | 0.000 | 0.890 | 0.471 | 0.374 | 0.560 |
| | T-Coffee | 0.643 | 0.971 | 0.867 | 0.107 | 0.864 | 0.000 | 0.860 | 0.464 | 0.370 | 0.530 |
| | | SP* | | | | | TC | | | | |
| RV60_4 | CLUSTALW | 0.097 | 0.716 | 0.409 | 0.238 | 0.482 | 0.000 | 0.530 | 0.095 | 0.210 | 0.000 |
| | CLUSTAL O. | 0.077 | **0.946** | 0.502 | 0.289 | 0.488 | 0.000 | **0.860** | **0.254** | 0.328 | 0.045 |
| | DIALIGN-TX | 0.120 | 0.841 | 0.451 | 0.241 | 0.452 | 0.000 | 0.580 | 0.128 | 0.222 | 0.000 |
| | MAFFT | 0.078 | 0.870 | 0.559 | 0.283 | **0.696** | 0.000 | 0.620 | 0.217 | 0.246 | **0.080** |
| | MUSCLE | 0.052 | 0.878 | 0.428 | 0.246 | 0.389 | 0.000 | 0.490 | 0.045 | 0.148 | 0.000 |
| | POA | 0.187 | 0.767 | 0.481 | 0.213 | 0.539 | 0.000 | 0.530 | 0.179 | 0.240 | 0.000 |
| | Probalign | **0.230** | 0.875 | **0.614** | 0.215 | 0.613 | 0.000 | 0.610 | 0.211 | 0.259 | 0.000 |
| | Probcons | 0.180 | 0.874 | 0.562 | 0.238 | 0.592 | 0.000 | 0.610 | 0.145 | 0.251 | 0.000 |
| | T-Coffee | 0.145 | 0.851 | 0.538 | 0.249 | 0.588 | 0.000 | 0.610 | 0.175 | 0.224 | 0.070 |
| | | SP* | | | | | TC* | | | | |
| RV70 | CLUSTALW | 0.354 | 0.906 | 0.717 | 0.211 | 0.817 | 0.000 | 0.610 | 0.296 | 0.261 | 0.330 |
| | CLUSTAL O. | 0.492 | 0.961 | 0.810 | 0.159 | **0.882** | 0.020 | 0.700 | **0.376** | 0.270 | 0.370 |
| | Dialign-TX | 0.339 | 0.884 | 0.712 | 0.219 | 0.836 | 0.000 | 0.590 | 0.290 | 0.241 | 0.335 |
| | MAFFT | 0.531 | **0.963** | 0.810 | 0.136 | 0.842 | **0.050** | **0.750** | 0.370 | 0.252 | 0.345 |
| | MUSCLE | 0.500 | 0.950 | 0.787 | 0.146 | 0.840 | 0.000 | 0.670 | 0.316 | 0.246 | 0.290 |
| | POA | 0.448 | 0.902 | 0.720 | 0.153 | 0.798 | 0.000 | 0.560 | 0.265 | 0.209 | 0.225 |
| | Probalign | 0.529 | 0.934 | 0.798 | 0.133 | 0.860 | 0.000 | 0.670 | 0.345 | 0.242 | **0.380** |
| | Probcons | **0.575** | 0.942 | **0.812** | 0.124 | 0.861 | **0.050** | 0.670 | 0.365 | 0.225 | 0.375 |
| | T-Coffee | 0.526 | 0.940 | 0.805 | 0.137 | 0.860 | **0.050** | 0.670 | 0.360 | 0.236 | 0.350 |

| | | Minimum | Maximum | Average | Standard Deviation | Median | Minimum | Maximum | Average | Standard Deviation | Median |
|---|---|---|---|---|---|---|---|---|---|---|---|
| | | **SP*** | | | | | **TC** | | | | |
| **RV911** | **CLUSTALW** | 0.000 | **1,000** | 0.482 | 0.377 | 0.400 | 0.000 | **1,000** | 0.261 | 0.425 | 0.000 |
| | **CLUSTAL O.** | 0.000 | **1,000** | 0.612 | 0.367 | 0.750 | 0.000 | **1,000** | 0.362 | 0.451 | 0.000 |
| | **Dialign-TX** | 0.000 | **1,000** | 0.556 | 0.389 | 0.629 | 0.000 | **1,000** | 0.305 | 0.463 | 0.000 |
| | **MAFFT** | 0.000 | **1,000** | 0.709 | 0.309 | 0.833 | 0.000 | **1,000** | 0.410 | 0.453 | 0.000 |
| | **MUSCLE** | 0.000 | **1,000** | 0.658 | 0.356 | 0.750 | 0.000 | **1,000** | 0.361 | 0.453 | 0.000 |
| | **POA** | 0.000 | **1,000** | 0.542 | 0.403 | 0.705 | 0.000 | **1,000** | 0.288 | 0.414 | 0.000 |
| | **Probalign** | 0.000 | **1,000** | 0.647 | 0.347 | 0.743 | 0.000 | **1,000** | 0.361 | 0.448 | 0.000 |
| | **Probcons** | **0.018** | **1,000** | **0.726** | 0.299 | 0.844 | 0.000 | **1,000** | **0.442** | 0.451 | **0.500** |
| | **T-Coffee** | 0.000 | **1,000** | **0.726** | 0.316 | **0.846** | 0.000 | **1,000** | 0.438 | 0.448 | **0.500** |
| | | **SP*** | | | | | **TC*** | | | | |
| **RV912** | **CLUSTALW** | 0.000 | **1,000** | 0.745 | 0.378 | **1,000** | 0.000 | **1,000** | 0.678 | 0.451 | **1,000** |
| | **CLUSTAL O.** | 0.000 | **1,000** | 0.789 | 0.336 | **1,000** | 0.000 | **1,000** | 0.698 | 0.453 | **1,000** |
| | **DIALIGN-TX** | 0.028 | **1,000** | 0.764 | 0.339 | **1,000** | 0.000 | **1,000** | 0.635 | 0.466 | **1,000** |
| | **MAFFT** | 0.265 | **1,000** | 0.891 | 0.204 | **1,000** | 0.000 | **1,000** | 0.767 | 0.396 | **1,000** |
| | **MUSCLE** | 0.300 | **1,000** | 0.892 | 0.189 | **1,000** | 0.000 | **1,000** | 0.772 | 0.379 | **1,000** |
| | **POA** | 0.000 | **1,000** | 0.675 | 0.415 | 0.981 | 0.000 | **1,000** | 0.589 | 0.473 | 0.940 |
| | **Probalign** | 0.013 | **1,000** | 0.870 | 0.245 | **1,000** | 0.000 | **1,000** | 0.736 | 0.436 | **1,000** |
| | **Probcons** | **0.300** | **1,000** | **0.931** | 0.156 | **1,000** | 0.000 | **1,000** | **0.836** | 0.353 | **1,000** |
| | **T-Coffee** | **0.300** | **1,000** | **0.931** | 0.156 | **1,000** | 0.000 | **1,000** | **0.836** | 0.353 | **1,000** |
| | | **SP*** | | | | | **TC*** | | | | |
| **RV913** | **CLUSTALW** | 0.294 | **1,000** | 0.927 | 0.156 | **1,000** | 0.000 | **1,000** | 0.818 | 0.334 | **1,000** |
| | **CLUSTAL O.** | 0.206 | **1,000** | 0.868 | 0.244 | **1,000** | 0.000 | **1,000** | 0.759 | 0.417 | **1,000** |
| | **DIALIGN-TX** | 0.009 | **1,000** | 0.826 | 0.314 | **1,000** | 0.000 | **1,000** | 0.749 | 0.415 | **1,000** |
| | **MAFFT** | 0.294 | **1,000** | 0.916 | 0.188 | **1,000** | 0.000 | **1,000** | 0.839 | 0.326 | **1,000** |
| | **MUSCLE** | 0.294 | **1,000** | 0.927 | 0.176 | **1,000** | 0.000 | **1,000** | 0.851 | 0.319 | **1,000** |
| | **POA** | 0.000 | **1,000** | 0.770 | 0.369 | **1,000** | 0.000 | **1,000** | 0.710 | 0.435 | **1,000** |
| | **Probalign** | 0.294 | **1,000** | 0.956 | 0.140 | **1,000** | 0.000 | **1,000** | 0.916 | 0.207 | **1,000** |
| | **Probcons** | **0.511** | **1,000** | **0.968** | 0.099 | **1,000** | 0.000 | **1,000** | **0.932** | 0.204 | **1,000** |
| | **T-Coffee** | **0.511** | **1,000** | 0.964 | 0.100 | **1,000** | 0.000 | **1,000** | 0.920 | 0.208 | **1,000** |
| | | **SP** | | | | | **TC** | | | | |
| **RV921** | **CLUSTALW** | 0.060 | **1,000** | 0.617 | 0.353 | 0.600 | 0.000 | **1,000** | 0.213 | 0.409 | 0.000 |
| | **CLUSTAL O.** | 0.000 | **1,000** | 0.643 | 0.371 | 0.751 | 0.000 | **1,000** | 0.399 | 0.474 | 0.000 |
| | **DIALIGN-TX** | 0.000 | **1,000** | 0.568 | 0.435 | 0.835 | 0.000 | **1,000** | 0.290 | 0.456 | 0.000 |
| | **MAFFT** | 0.320 | **1,000** | 0.822 | 0.233 | 0.929 | 0.000 | **1,000** | 0.538 | 0.430 | 0.500 |
| | **MUSCLE** | 0.003 | **1,000** | 0.727 | 0.389 | 0.929 | 0.000 | **1,000** | 0.544 | 0.408 | 0.750 |
| | **POA** | 0.000 | **1,000** | 0.557 | 0.446 | 0.784 | 0.000 | **1,000** | 0.306 | 0.421 | 0.000 |
| | **Probalign** | 0.000 | **1,000** | 0.819 | 0.335 | **0.935** | 0.000 | **1,000** | **0.597** | 0.444 | **0.770** |
| | **Probcons** | **0.527** | **1,000** | **0.881** | 0.163 | **0.935** | 0.000 | **1,000** | 0.571 | 0.425 | 0.750 |
| | **T-Coffee** | 0.133 | **1,000** | 0.829 | 0.253 | 0.883 | 0.000 | **1,000** | 0.556 | 0.424 | 0.660 |
| | | **SP** | | | | | **TC** | | | | |
| **RV922** | **CLUSTALW** | 0.000 | **1,000** | 0.602 | 0.347 | 0.632 | 0.000 | **1,000** | 0.198 | 0.397 | 0.000 |
| | **CLUSTAL O.** | 0.000 | **1,000** | 0.666 | 0.360 | 0.803 | 0.000 | **1,000** | 0.371 | 0.468 | 0.000 |
| | **DIALIGN-TX** | 0.000 | **1,000** | 0.595 | 0.424 | 0.847 | 0.000 | **1,000** | 0.282 | 0.439 | 0.000 |
| | **MAFFT** | **0.320** | **1,000** | 0.801 | 0.236 | 0.877 | 0.000 | **1,000** | 0.501 | 0.416 | 0.435 |
| | **MUSCLE** | 0.095 | **1,000** | 0.739 | 0.329 | 0.925 | 0.000 | **1,000** | 0.476 | 0.459 | 0.550 |
| | **POA** | 0.000 | **1,000** | 0.578 | 0.435 | 0.814 | 0.000 | **1,000** | 0.284 | 0.413 | 0.000 |
| | **Probalign** | 0.000 | **1,000** | 0.823 | 0.319 | **0.932** | 0.000 | **1,000** | **0.539** | 0.452 | **0.660** |
| | **Probcons** | 0.121 | **1,000** | 0.819 | 0.251 | 0.898 | 0.000 | **1,000** | 0.487 | 0.458 | 0.625 |
| | **T-Coffee** | 0.133 | **1,000** | **0.841** | 0.242 | 0.909 | 0.000 | **1,000** | 0.516 | 0.433 | 0.630 |
| | | **SP*** | | | | | **TC** | | | | |
| **RV931** | **CLUSTALW** | 0.000 | **1,000** | 0.527 | 0.363 | 0.600 | 0.000 | **1,000** | 0.184 | 0.382 | 0.000 |
| | **CLUSTAL O.** | 0.000 | **1,000** | 0.507 | 0.394 | 0.600 | 0.000 | **1,000** | 0.236 | 0.422 | 0.000 |
| | **DIALIGN-TX** | 0.000 | **1,000** | 0.484 | 0.389 | 0.397 | 0.000 | **1,000** | 0.208 | 0.415 | 0.000 |
| | **MAFFT** | 0.012 | **1,000** | 0.645 | 0.307 | 0.725 | 0.000 | **1,000** | 0.239 | 0.424 | 0.000 |
| | **MUSCLE** | 0.000 | **1,000** | 0.662 | 0.332 | 0.790 | 0.000 | **1,000** | 0.302 | 0.440 | 0.000 |
| | **POA** | 0.000 | **1,000** | 0.418 | 0.382 | 0.279 | 0.000 | **1,000** | 0.192 | 0.384 | 0.000 |
| | **Probalign** | 0.000 | **1,000** | 0.637 | 0.382 | 0.804 | 0.000 | **1,000** | 0.355 | 0.472 | 0.000 |
| | **Probcons** | **0.028** | **1,000** | **0.717** | 0.320 | 0.846 | 0.000 | **1,000** | 0.369 | 0.465 | 0.000 |
| | **T-Coffee** | **0.028** | **1,000** | 0.698 | 0.357 | **0.849** | 0.000 | **1,000** | **0.425** | 0.480 | 0.000 |
| | | **SP** | | | | | **TC** | | | | |
| **RV932** | **CLUSTALW** | 0.000 | **1,000** | 0.439 | 0.351 | 0.457 | 0.000 | **1,000** | 0.178 | 0.380 | 0.000 |
| | **CLUSTAL O.** | 0.000 | **1,000** | 0.464 | 0.435 | 0.288 | 0.000 | **1,000** | 0.285 | 0.454 | 0.000 |
| | **DIALIGN-TX** | 0.000 | **1,000** | 0.453 | 0.382 | 0.361 | 0.000 | **1,000** | 0.217 | 0.391 | 0.000 |
| | **MAFFT** | 0.012 | **1,000** | 0.623 | 0.367 | 0.749 | 0.000 | **1,000** | 0.314 | 0.458 | 0.000 |
| | **MUSCLE** | 0.000 | **1,000** | 0.564 | 0.361 | 0.706 | 0.000 | **1,000** | 0.253 | 0.406 | 0.000 |
| | **POA** | 0.000 | **1,000** | 0.393 | 0.376 | 0.200 | 0.000 | **1,000** | 0.192 | 0.384 | 0.000 |
| | **Probalign** | 0.000 | **1,000** | 0.489 | 0.414 | 0.379 | 0.000 | **1,000** | 0.267 | 0.433 | 0.000 |
| | **Probcons** | 0.000 | **1,000** | **0.644** | 0.383 | **0.801** | 0.000 | **1,000** | **0.355** | 0.473 | 0.000 |
| | **T-Coffee** | **0.028** | **1,000** | 0.602 | 0.381 | 0.763 | 0.000 | **1,000** | 0.333 | 0.458 | 0.000 |
| | | **SP*** | | | | | **TC*** | | | | |
| **RV941** | **CLUSTALW** | 0.000 | **1,000** | 0.519 | 0.328 | 0.536 | 0.000 | **1,000** | 0.147 | 0.337 | 0.000 |
| | **CLUSTAL O.** | 0.000 | **1,000** | 0.610 | 0.373 | 0.736 | 0.000 | **1,000** | 0.293 | 0.447 | 0.000 |
| | **DIALIGN-TX** | 0.000 | **1,000** | 0.561 | 0.369 | 0.640 | 0.000 | **1,000** | 0.210 | 0.393 | 0.000 |
| | **MAFFT** | 0.012 | **1,000** | 0.718 | 0.283 | 0.742 | 0.000 | **1,000** | 0.366 | 0.425 | 0.000 |
| | **MUSCLE** | 0.000 | **1,000** | 0.689 | 0.309 | 0.771 | 0.000 | **1,000** | 0.318 | 0.430 | 0.000 |
| | **POA** | 0.000 | **1,000** | 0.454 | 0.345 | 0.535 | 0.000 | **1,000** | 0.143 | 0.319 | 0.000 |
| | **Probalign** | 0.000 | **1,000** | 0.715 | 0.333 | 0.836 | 0.000 | **1,000** | 0.425 | 0.472 | 0.000 |
| | **Probcons** | **0.093** | **1,000** | **0.811** | 0.260 | **0.954** | 0.000 | **1,000** | **0.519** | 0.457 | 0.650 |
| | **T-Coffee** | 0.040 | **1,000** | 0.797 | 0.278 | 0.949 | 0.000 | **1,000** | 0.517 | 0.474 | **0.695** |
| | | **SP*** | | | | | **TC** | | | | |
| **RV942** | **CLUSTALW** | 0.000 | **1,000** | 0.524 | 0.353 | 0.536 | 0.000 | **1,000** | 0.173 | 0.356 | 0.000 |
| | **CLUSTAL O.** | 0.000 | **1,000** | 0.497 | 0.356 | 0.542 | 0.000 | **1,000** | 0.208 | 0.394 | 0.000 |
| | **DIALIGN-TX** | 0.000 | **1,000** | 0.518 | 0.385 | 0.559 | 0.000 | **1,000** | 0.210 | 0.393 | 0.000 |
| | **MAFFT** | 0.000 | **1,000** | 0.663 | 0.325 | 0.682 | 0.000 | **1,000** | 0.356 | 0.446 | 0.000 |
| | **MUSCLE** | 0.000 | **1,000** | 0.619 | 0.328 | 0.696 | 0.000 | **1,000** | 0.238 | 0.374 | 0.000 |
| | **POA** | 0.000 | **1,000** | 0.446 | 0.344 | 0.525 | 0.000 | **1,000** | 0.143 | 0.319 | 0.000 |
| | **Probalign** | 0.000 | **1,000** | 0.633 | 0.391 | 0.763 | 0.000 | **1,000** | 0.344 | 0.462 | 0.000 |
| | **Probcons** | 0.000 | **1,000** | 0.709 | 0.357 | 0.868 | 0.000 | **1,000** | 0.439 | 0.471 | 0.100 |
| | **T-Coffee** | **0.036** | **1,000** | **0.796** | 0.283 | **0.936** | 0.000 | **1,000** | **0.505** | 0.471 | **0.695** |
