# Supplementary material for: Assessing the efficiency of multiple sequence alignment programs
Source: Algorithms Mol Biol. 2014 Mar 6;9:4. doi: 10.1186/1748-7188-9-4 (PMC4015676; doi:10.1186/1748-7188-9-4)
Supplement: Additional file 3 — Overview of computational cost from execution time measure for BAliBASE Reference sets 1–7 and 9. Execution times are presented for each program, reference set and sequence type (full-length or truncated), when available. Values in bold are the smallest found. Execution times over 2.5 hours were not applicable (N/A) in this study. [file 1748-7188-9-4-S3.pdf]

Execution time										
	Program									
Reference set	CLUSTALW	CLUSTAL OMEGA	DIALIGN-TX	MAFFT	MUSCLE	POA	Probalign	Probcons	T-Coffee	T-Coffee multi-core
RV11 - BB	**0m7.498s**	1m1.334s	0m27.187s	0m26.634s	0m24.251s	0m9.545s	0m42.088s	1m7.522s	1m25.678s	0m27.141s
RV11 - BBS	**0m4.976s**	0m31.740s	0m21.263s	0m16.381s	0m13.374s	0m6.107s	0m23.209s	0m39.501s	0m57.073s	0m20.858s
RV12 - BB	**0m26.066s**	2m50.002s	1m11.603s	0m46.264s	0m32.850s	0m27.318s	3m13.357s	4m3.050s	4m58.272s	0m55.112s
RV12 - BBS	0m17.093s	1m16.247s	0m54.649s	0m26.654s	**0m15.618s**	0m17.170s	1m51.798s	2m25.186s	3m21.004s	0m37.936s
RV20 - BB	5m35.036s	13m50.509s	25m25.649s	9m50.739s	**4m18.717s**	5m28.056s	69m31.247s	86m10.229s	N/A	11m36.657s
RV20 - BBS	2m40.040s	3m42.444s	15m58.439s	2m56.359s	**1m1.571s**	2m4.473s	23m46.344s	31m17.809s	N/A	5m24.666s
RV30 - BB	6m57.093s	12m17.222s	35m38.795s	9m44.845s	**4m26.213s**	8m17.887s	105m9.562s	135m43.204s	N/A	23m29.983s
RV30 - BBS	3m30.659s	4m11.303s	23m25.755s	3m51.256s	**1m35.370s**	3m38.860s	43m28.312s	55m35.653s	N/A	14m53.200s
RV40	**4m8.404s**	30m1.639s	15m37.065s	10m55.672s	9m39.285s	6m25.430s	57m34.619s	78m42.505s	N/A	10m30.121s
RV50 - BB	**1m30.474s**	7m20.438s	5m31.331s	4m58.053s	2m13.417s	2m14.645s	22m49.759s	30m13.576s	63m24.957s	3m39.789s
RV50 - BBS	0m38.491s	1m58.127s	2m52.437s	1m20.104s	**0m32.283s**	0m45.668s	6m24.013s	8m56.301s	34m45.827s	1m42.674s
RV60_1a	0m5.262s	0m29.075s	0m13.674s	0m5.676s	**0m4.415s**	0m5.667s	1m3.495s	1m10.823s	0m57.323s	N/A
RV60_1b	**0m3.393s**	0m28.845s	0m11.134s	0m7.335s	0m5.741s	0m3.978s	0m32.672s	0m39.188s	0m36.146s	N/A
RV60_2a	**0m13.521s**	1m15.739s	5m27.936s	0m19.522s	0m34.224s	0m23.410s	3m52.679s	5m27.935s	N/A	2m4.105s
RV60_2b	**0m8.373s**	0m47.936s	6m40.574s	0m22.352s	0m34.899s	0m10.445s	1m56.721s	2m21.670s	74m9.966s	N/A
RV60_2c	**0m17.187s**	2m17.230s	4m59.946s	1m4.091s	1m19.117s	0m20.961s	3m5.485s	4m5.804s	37m15.232s	N/A
RV60_3	0m20.143s	1m33.852s	7m54.315s	0m31.294s	1m12.873s	**0m17.057s**	4m45.675s	6m7.723s	N/A	1m42.156s
RV60_4	**0m15.946s**	2m28.757s	2m50.660s	1m3.929s	1m36.620s	0m19.797s	2m38.414s	3m26.853s	33m16.131s	N/A
RV70	2m52.749s	6m35.009s	11m52.401s	6m0.906s	**1m49.849s**	3m31.549s	44m53.260s	55m37.415s	N/A	9m29.643s
RV911	**2m50.399s**	24m38.417s	9m37.420s	8m41.313s	7m27.611s	5m6.268s	48m33.701s	61m41.440s	105m2.546s	N/A
RV912	**0m15.694s**	1m35.747s	0m40.769s	0m28.474s	0m18.222s	0m15.996s	2m0.827s	2m27.454s	2m49.669s	N/A
RV913	0m40.505s	1m53.081s	2m13.139s	0m34.556s	**0m16.553s**	0m26.253s	7m52.507s	8m34.256s	13m25.391s	N/A
RV921	**1m57.086s**	8m1.870s	7m19.800s	5m19.175s	2m45.307s	2m42.569s	31m1.909s	37m45.279s	114m48.969s	N/A
RV922	**2m8.015s**	9m5.021s	8m58.422s	5m29.722s	4m38.607s	3m5.030s	34m21.711s	42m56.034s	N/A	5m40.043s
RV931	4m0.502s	8m10.592s	17m24.926s	3m49.859s	**2m24.695s**	4m34.612s	66m1.412s	75m8.925s	N/A	19m6.800s
RV932	**10m7.277s**	40m20.063s	45m53.084s	15m5.090s	20m13.858s	16m20.858s	N/A	N/A	N/A	38m38.261s
RV941	15m10.005s	39m26.377s	79m5.863s	**11m25.099s**	20m6.703s	28m29.998s	N/A	N/A	N/A	65m35.516s
RV942	**22m32.953s**	75m38.017s	120m31.877s	38m12.789s	49m56.348s	45m32.788s	N/A	N/A	N/A	99m33.435s
