# Supplementary material for: Assessing the efficiency of multiple sequence alignment programs
Source: Algorithms Mol Biol. 2014 Mar 6;9:4. doi: 10.1186/1748-7188-9-4 (PMC4015676; doi:10.1186/1748-7188-9-4)
Supplement: Additional file 4 — Overview of computational costs from memory usage measure from BAliBASE Reference sets 1–7 and 9. Memory usage (kb) are presented for each program, reference set and sequence type (full-length or truncated), when available. Values in bold are the smallest found. For programs where execution times exceeded 2.5 hours, memory usage was not applicable (N/A) in this study. [file 1748-7188-9-4-S4.pdf]

Memory usage										
	Program									
Reference set	CLUSTALW	CLUSTAL OMEGA	DIALIGN-TX	MAFFT	MUSCLE	POA	Probalign	Probcons	T-Coffee	T-Coffee multi-core
**RV11 - BB**	**920**	63200	6416	152092	17772	6016	12408	30600	98564	890936
**RV11 - BBS**	**744**	26580	5956	136968	14072	4080	7748	18368	56736	560124
**RV12 - BB**	**2064**	495644	8100	149912	28388	12808	45476	110312	377104	2417580
**RV12 - BBS**	**1032**	71168	7484	141880	18388	9476	20956	67172	174116	2235284
**RV20 - BB**	**2292**	233688	31840	221580	42040	31716	127088	131120	N/A	3264196
**RV20 - BBS**	**1072**	31248	26376	142800	30969	8900	82644	74768	N/A	984908
**RV30 - BB**	**2556**	158836	42320	189332	49976	27608	481456	381860	N/A	1418880
**RV30 - BBS**	**1480**	26596	42492	151576	40056	15260	390740	300728	N/A	1017692
**RV40**	**2636**	1305324	27764	581304	86044	164696	338600	538284	N/A	3542472
**RV50 - BB**	**2896**	406900	22096	329304	58888	46732	152580	212424	619696	2761500
**RV50 - BBS**	**1452**	72040	16544	156264	28288	11616	79704	63844	173052	1129424
**RV60_1a**	**1200**	117572	10360	123220	21200	14320	36148	91804	297340	N/A
**RV60_1b**	**1192**	168012	7648	121120	19460	12232	34296	84740	290332	N/A
**RV60_2a**	**1636**	72212	37972	151424	31408	13192	176076	165108	N/A	835424
**RV60_2b**	**1268**	62088	11180	153544	23024	6948	43240	39464	123296	N/A
**RV60_2c**	**3208**	543260	10088	222712	57824	27940	67052	127936	463604	N/A
**RV60_3**	**4156**	645536	33844	183328	66684	12944	70984	153776	N/A	2957388
**RV60_4**	**1796**	573380	19100	162240	31836	20540	95892	221064	793388	N/A
**RV70**	**2608**	220972	48584	220216	55164	50820	328164	246968	N/A	1435660
**RV911**	**3688**	809752	33208	392160	98772	155120	263572	475536	691532	N/A
**RV912**	**1052**	103888	11932	144452	18764	8232	26688	63588	223020	N/A
**RV913**	**1328**	123580	16748	116340	33848	11340	51392	102152	331636	N/A
**RV921**	**2344**	445512	31500	274860	64268	52320	208832	266676	869784	N/A
**RV922**	**2552**	502400	32840	261288	66368	63544	211104	302976	N/A	5368980
**RV931**	**2444**	334612	110076	181284	73112	50940	713440	541752	N/A	6931724
**RV932**	**7572**	435796	115636	380868	137668	151464	N/A	N/A	N/A	5771668
**RV941**	**3560**	1033776	228244	544480	227004	208980	N/A	N/A	N/A	2710852
**RV942**	**14716**	1310652	265912	497056	262032	478936	N/A	N/A	N/A	4161148
